# A quantitative high-resolution computational mechanics cell model for growing and regenerating tissues

**DOI:** 10.1007/s10237-019-01204-7

**Published:** 2019-11-20

**Authors:** Paul Van Liedekerke, Johannes Neitsch, Tim Johann, Enrico Warmt, Ismael Gonzàlez-Valverde, Stefan Hoehme, Steffen Grosser, Josef Kaes, Dirk Drasdo

**Affiliations:** 1 Inria Paris & Sorbonne Université LJLL, 2 Rue Simone IFF, 75012 Paris, France; 2 IfADo - Leibniz Research Centre for Working Environment and Human Factors, Ardeystrasse 67, Dortmund, Germany; 3 Interdisciplinary Centre for Bioinformatics, Leipzig University, Härtelstr. 16–18, 04107 Leipzig, Germany; 4 Institute for Computer Science, Leipzig University, Härtelstr. 16–18, 04107 Leipzig, Germany; 5 Faculty of Physics and Earth Science, Peter Debye Institute for Soft Matter Physics, Leipzig University, Linnéstraße 5, 04103 Leipzig, Germany; 6 M2be, University of Zaragoza, C/ Maria de Luna s/n, 50018 Zaragoza, Spain

**Keywords:** Cell-based model, High resolution cell model, Cell mechanics, Liver regeneration, Optical stretcher

## Abstract

Mathematical models are increasingly designed to guide experiments in biology, biotechnology, as well as to assist in medical decision making. They are in particular important to understand emergent collective cell behavior. For this purpose, the models, despite still abstractions of reality, need to be quantitative in all aspects relevant for the question of interest. This paper considers as showcase example the regeneration of liver after drug-induced depletion of hepatocytes, in which the surviving and dividing hepatocytes must squeeze in between the blood vessels of a network to refill the emerged lesions. Here, the cells’ response to mechanical stress might significantly impact the regeneration process. We present a 3D high-resolution cell-based model integrating information from measurements in order to obtain a refined and quantitative understanding of the impact of cell-biomechanical effects on the closure of drug-induced lesions in liver. Our model represents each cell individually and is constructed by a discrete, physically scalable network of viscoelastic elements, capable of mimicking realistic cell deformation and supplying information at subcellular scales. The cells have the capability to migrate, grow, and divide, and the nature and parameters of their mechanical elements can be inferred from comparisons with optical stretcher experiments. Due to triangulation of the cell surface, interactions of cells with arbitrarily shaped (triangulated) structures such as blood vessels can be captured naturally. Comparing our simulations with those of so-called center-based models, in which cells have a largely rigid shape and forces are exerted between cell centers, we find that the migration forces a cell needs to exert on its environment to close a tissue lesion, is much smaller than predicted by center-based models. To stress generality of the approach, the liver simulations were complemented by monolayer and multicellular spheroid growth simulations. In summary, our model can give quantitative insight in many tissue organization processes, permits hypothesis testing in silico, and guide experiments in situations in which cell mechanics is considered important.

**Electronic supplementary material:**

The online version of this article (10.1007/s10237-019-01204-7) contains supplementary material, which is available to authorized users.

## Introduction

Driven by the insight that multicellular organization cannot be explained from the viewpoint of biochemical processes alone and flanked by recent development of methods in imaging and probing of physical forces at small scales, the role of mechanics in the interplay of cell and multicellular dynamics is moving into the main focus of biological research (Fletcher and Mullins 2010). Cells respond on mechanical stress both passively and actively; hence, an understanding of the growth and division behavior of proliferating cells is not possible without properly taking into account the mechanical components underlying these processes. Mathematical models are being established as an additional cornerstone to interpret biological observations and provide information to clinicians entering in their decisions (Rodriguez et al. 2013; Karolak et al. 2018). This requires high reliability of models and quantitative simulations.

A clinical relevant example is the regeneration of liver after drug-induced toxic damage after paracetamol (acetaminophen, APAP) or tetrachloride (CCl4) overdose. These drugs generate a characteristic central necrotic hepatocyte-depleted lesion in each liver lobule, which is the smallest repetitive functional and anatomical unit of liver. Hoehme et al. (2010) used confocal laser scanning micrographs to set up a realistic spatial-temporal agent-based model of a liver lobule. In their model, hepatocytes were represented as individual units (agents) parameterized by biophysical and biological quantities and able to move as a consequence of forces on the cell, and of the cells’ own micro-motility. The cells were approximated as spheres (the shape a cell adopts in isolation), while the forces between them are simulated as forces between the cell centers, which is why these models are often termed “center-based model” (CBM). Center-based models have proven useful to mimic tissue organization processes, for example, in vitro and in early development (see, e.g., Drasdo and Höhme 2005; Drasdo et al. 2007; Geris et al. 2010; Buske et al. 2011) and have been shown to provide a good framework for multiscale simulations in tumor development (e.g., Ramis-Conde et al. 2009; Delile et al. 2017). For liver, the CBM predicted that active uniform micro-motility forces would not suffice to close the characteristic necrotic tissue lesions generated in the center of each liver lobule but further mechanisms as directed migration and oriented cell division along closest micro-vessels (named hepatocyte-sinusoid alignment, HSA) would be necessary to explain the observed regeneration scenarios. HSA has been subsequently be experimentally validated. To obtain these results, extensive simulated sensitivity analyses had been performed varying each parameter of the model within its physiological range (Drasdo et al. 2014). In order to arrive at such conclusions, the model must for a given set of parameters be able to realistically and quantitatively predict the outcome of the regeneration process. The model can then be viewed as a mapping from a set of parameters to a set of macroscopic observables such as the size of the necrotic lesion or the cell density in the lobule.

A major drawback of center-based models is that they are based on the calculation of pairwise forces (usually Hertz force, Johnson–Kendall–Roberts force or related) between cell centers, which lack accuracy in dense cell aggregates under compression where the interaction force of a cell with one neighbor impacts on its interaction force with another neighbor. A consequence of a naive use of pairwise forces is the absence of a notion of cell volume. Hence, simulations with quasi-incompressible cells characterized by a Poisson ratio of $\nu \approx 0.5$ can lead to unrealistic multicellular arrangements and thus to false predictions. Such situation may occur during liver regeneration after APAP or CCl4 intoxication where many cells enter the cell cycle almost at the same time close the drug-induced lesion. It also occurs in the interior of growing multicellular spheroids. Model corrections have been proposed to circumvent this shortcoming, but so far a fully consistent approach for center-based models has been out of reach as this requires to consistently relate cell–cell interaction forces and cell shape. In center-based models, cell shape can only be estimated for very small cell deformations. Approximating cell shape by Voronoi-tessellation Schaller and Meyer-Hermann (2005) permits to calculate a cell volume, but the interaction forces are in most situations inconsistent with the cell–cell contact areas resulting from this tessellation (Van Liedekerke et al. 2015).

The shortcomings of the CBM call for a model type that consistently relates cell strain and stresses in cells within multicellular assemblies. A large category of models called lattice-free, force-based “deformable cell models” (DCMs) has been developed to meet these needs (Rejniak 2007; Sandersius and Newman 2008; Jamali et al. 2010; Odenthal et al. 2013; Van Liedekerke et al. 2019). Their lattice-based counterparts, called cellular Potts models (CPM), are popular in biomedical modeling, partially because of their straightforward implementation. The dynamics in CPM is principally stochastic in nature and is based on the minimization of energy functionals (Hamiltonian) and Monte Carlo sampling over a vast number of lattice sites (e.g., Graner and Glazier 1992; van Oers et al. 2014; Palm and Merks 2013). Force-based methods use equations of motion, which facilitates presenting both stochastic and deterministic components. Early work in this field has treated multicellular spheroids, various cellular patterns in developing ductal carcinoma in situ, invasive tumors as well as normal development of epithelial ductal monolayers and their various mutants (e.g., Galle et al. 2005, Rejniak 2007; Drasdo et al. 2007; Dillon et al. 2008; Rejniak and Anderson 2008; Sandersius and Newman 2008). Deformable models have also been developed to study the dynamics of erythrocytes in blood flow (Hosseini and Feng 2009; Fedosov et al. 2010; Van Liedekerke et al. 2013; Fedosov et al. 2011), cellular rheology (Sandersius et al. 2011), or even impact of tissue (Van Liedekerke et al. 2010, 2011). Recent approaches focus more and more on the explicit representation of subcellular details such as a nucleus, and cytoskeleton (Jamali et al. 2010; Chen et al. 2018). Other DCM types, such as the vertex model, focus intrinsically more on epithelial sheets (Fletcher et al. 2014).

In this paper, we present an agent-based, high-resolution deformable cell model in three dimensions allowing to simulate cell growth and tissues. The model is particularly suited to study the interplay between cell growth, mechanical variables and tissue architecture and is here employed to mimic tissue regeneration in a part of a liver lobule. Our cell model builds upon earlier work by Odenthal et al. (2013) and Van Liedekerke et al. (2019), a DCM type whereby the cell surface is triangulated and the nodes are connected by viscoelastic elements, representing the cell membrane and actin cortical cytoskeleton and homogeneous cytoplasm. In the work of Odenthal et al., it was shown that this DCM could quantitatively mimic the adhesion dynamics of red blood cells on a surface, yet cell growth and division were not envisaged. Here, we enrich the model with those features, enabling us to model various multicellular systems. DCM types with cell division capabilities have been created by a number of authors (e.g., Jamali et al. 2010; Tanaka et al. 2015; Milde et al. 2014), whereby in Jamali et al. (2010) the mechanical processes leading to cytokinesis have been explicitly mimicked. Most of these models are two-dimensional. Cytokinesis, during which a cell splits into two separated daughter cells, completes the mitosis phase. Mitosis and cytokinesis together take about 1 h compared to the duration of the cell cycle $\sim 24$ h in most mammalian cells, hence are very short. Accurate simulation of the cytokinesis process in three-dimensional triangulated cells turns out to be challenging with regard to both the algorithms and computational efforts. For these reasons, our DCM^1^ mimics the cell division event in one step, yet ensuring that the daughter cells precisely fill the space of the mother cell.

Our model has basically two key advantages. First, the model can be parameterized by physical and biokinetic parameters. By direct comparison with optical stretcher experiments (Guck et al. 2001), which cause cell deformation as a response of an externally applied stress by a laser beam, we determine the type of the cell’s viscoelastic elements and the magnitude of its parameters. In addition to the basic cortical triangulated model, we have created the possibility for each cell to mimic an internal cytoskeleton by connecting the cell cortex and the cell nucleus by viscoelastic elements. However, as we have found that the deformation of cells can quantitatively be captured even without explicit representation of cell nuclei, we perform the simulations in this paper discerning only the cell boundary elements. Second, the model design is such that each cell can interact with any arbitrarily shaped objects that are either represented by a triangulated surface (rigid of flexible, see, for example, Fig. 11), which can also be generated by external specialized software, or represented by a mathematical surface. The model is not restricted to simulate elementary structures such as spheroids or monolayers. In liver architecture, relevant for our final application example, cells interact with other cells, but also with a complex network of micro-vessels (named liver sinusoids).

The approach facilitates performing hybrid tissue simulations where DCM cells can interact with center-based cells. Generally, hybrid simulations are useful if simulations of an entire tissue need to be performed in a reasonable time (González-Valverde and García-Aznar 2018). Hybrid modeling combining DCM and center-based model, conceptually similar to the hybrid strategy proposed in Kim et al. (2013), enables us to simulated part of the system as higher spatial resolution and thereby “zoom” into spatial substructures of interest. To ensure that the center-based model behaves “on average” as the DCM, which is a priori not the case due to the shortcomings of the CBM approach as discussed above, we here propose a simple correction scheme in which the interaction forces of the CBM are calibrated from simulations with the DCM. In this way, the DCM can be used to verify the systems behavior of the CBM for small cell populations, while the CBM can be used to simulate large cell populations. We demonstrate this by direct comparison of a CBM and a DCM in the same liver lobule.

This paper is structured as follows. The technical details of the DCM and the CBM (and force calibration) are explained in Sect. 2. Following, we first study the single cell dynamics of the DCM. Each model parameter, biomechanical and biokinetic, can be directly associated with a physical property, i.e., can either be directly measured or be calibrated by comparison to single cell or multicellular experiments. This makes it possible to identify physiological parameter ranges. We here compare directly optical stretcher real to in silico (with the DCM) experiments (Sect. 3.1.1) to identify the nature of viscoelastic elements in the DCM and their parameters. Next, we consider classical in silico experiments of two adhering cells being mechanically separated to identify the model parameters for cell–cell adhesion. We verify whether the contact forces and stress distribution on the cell surface predicted by our model are physically plausible (Sect. 3.1.2). These experiments can generally serve as means to calibrate the parameters of a single cell accurately. Secondly, we consider the growth and proliferation of the cells in the classical settings of growing monolayer and multicellular spheroids, which have been studied in numerous experiments and modeling works (Sect. 3.2). Finally, we perform simulations of regeneration dynamics after intoxication of liver with CCl4/APAP using the DCM and compare simulation results to both experimental data and simulation results with the CBM similar as in Hoehme et al. (2010). We analyze the results and basic differences in terms of dynamics and tissue architecture in Sect. 3.3. The cell shapes obtained by simulations with the DCM can in principle readily be compared with high-resolution confocal microscopy images (e.g., Morales-Navarrete et al. 2019). Together with developments in tissue clearing (Tainaka et al. 2016) this might open up the possibility to infer the stresses on the cells in full 3D volume reconstructions from laser scanning confocal micrographs of the cell shapes. Alternatively, the elastic properties of emergent tissues simulated with the model can be compared to elastographic images (Sack et al. 2013).

## Mathematical models

In this section, we define the specific agent-based mathematical models that we use later in specific applications. In agent-based models of multicellular assemblies, every cell ($=$ agent) is represented as an individual separated object that is able to move, grow, divide, and die. The cell can interact with other cells as well as other objects in its environment. As such, emerging effects of these many interactions can be studied. We explain the two types of single cell-based models used in this work: first the deformable cell model (DCM), and then we recapitulate the center-based model (CBM).

### Deformable cell model (DCM)

**Fig. 1:**
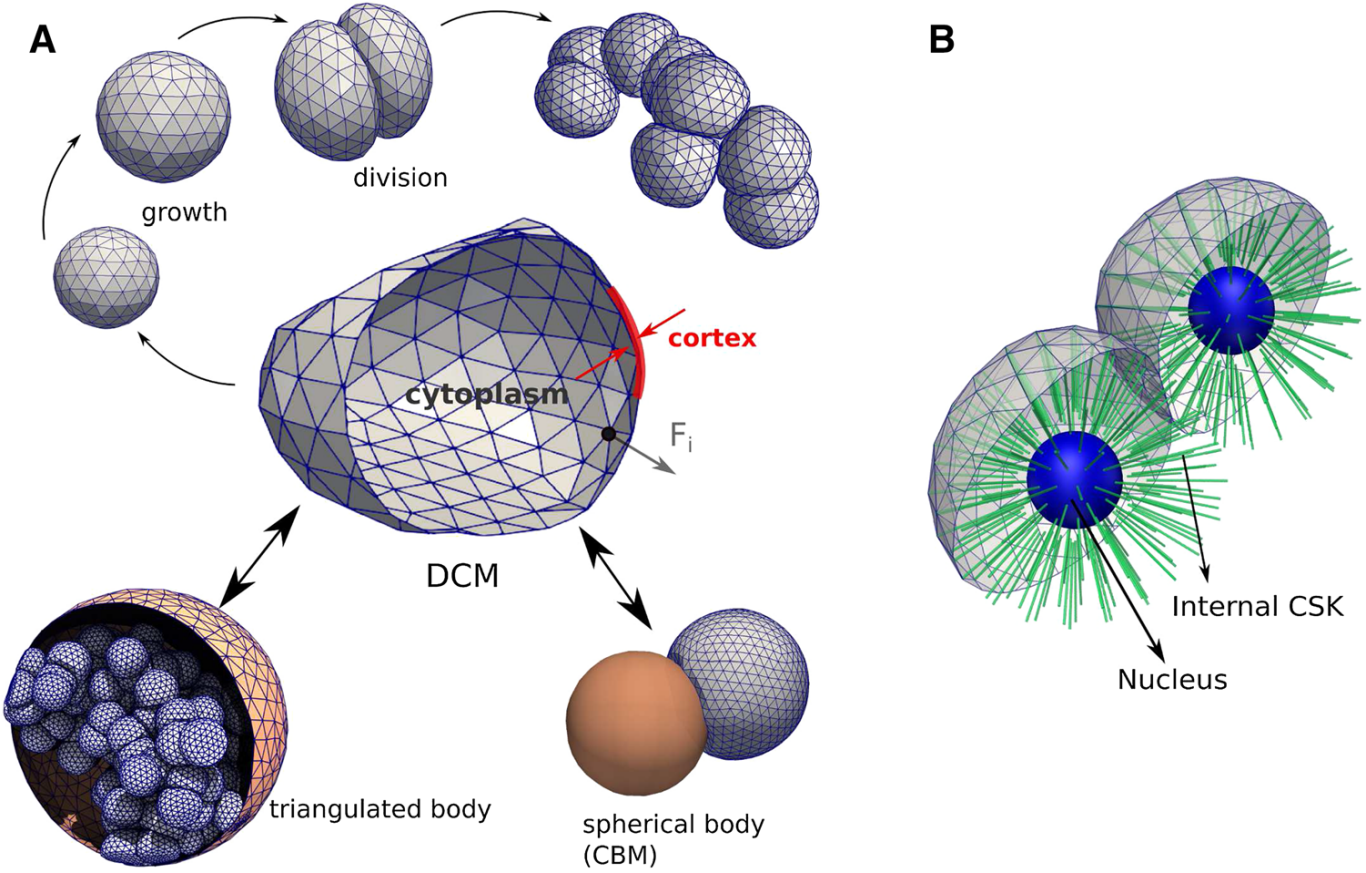
**a** The force-based deformable cell model (DCM) and its basic components and functionality used in this work. A cell is represented by a viscoelastic triangulated shell (cortex) containing a compressible cytoplasm (center). The cells can grow until they split into two new cells, eventually creating a clump of adhering cells (top). Each nodal point of the cell moves according to an equation of motion in response to a force ${\mathbf {F}}_{i}$. The cells can interact with rigid triangulated bodies (such as here a capsule encapsulating them) or simple geometric bodies such as a center-based model (bottom). **b** Same model showing prototype of the model where a nucleus and internal cytoskeleton is included explicitly

In our DCM, the cell surface is triangulated with viscoelastic elements along each edge of each triangle. This creates a global deformable structure with many degrees of freedom (Van Liedekerke et al. 2010; Odenthal et al. 2013; Van Liedekerke et al. 2019; Guyot et al. 2016). Throughout this paper, we do not represent the cell organelles separately, but by a homogeneous isotropic viscoelastic material (see Fig. 1a). Nevertheless, our model in principle permits the explicit representation of organelles, e.g., by triangulating them in the same way as the cell surface and connecting the structures by viscoelastic elements (Fig. 1b).

We found that the homogenized approximation sufficed as the explicit representation of cell organelles was both not needed and computationally costly for the questions studied in this paper. The viscoelastic elements and parameters of the cell surface are calibrated such that they simultaneously account for the mechanical response of the cell membrane and cell cortex, in particular the cortical cytoskeleton (CSK), i.e., forces that represent in-plane and bending elasticity of the cortex and the plasma membrane. Moreover, we account for a force combining contributions from the cytoplasm and the nucleus in response to cell compression. Cell–cell interactions induce external forces, which may be repulsive or adhesive, or both.

Cells in our model can migrate, die, grow, and divide when their volume has doubled. When a cell divides, two new cells are created that fit in the envelope of the mother cell and all aforementioned forces are automatically invoked on the daughter cells (see Fig. 3). Apoptosis can be modeled as well. The details of the model components are explained in the following sections.

#### Forces and equations of motion

Movement and deformation of a cell can be calculated from a force balance summarized in the following equation of motion for each node *i* of the triangulation:

1 $$\begin{aligned}&\underbrace{\sum _{j}{\varvec{\Gamma }}^{\mathrm{c}}_{\mathrm{nn},ij} (\mathbf {v}_i-\mathbf {v}_j)}_{\text {(1)}} +\underbrace{\sum _{k}{\varvec{\Gamma }}^{\mathrm{cc}}_{\mathrm{nn},ik} (\mathbf {v}_i-\mathbf {v}_k)}_{\text {(2)}} +\underbrace{{\varvec{\Gamma }}_{\mathrm{ns},i} \mathbf {v}_i}_{\text {(3)}} \nonumber \\&\quad = \underbrace{\sum _{j}{\mathbf {F}}_{\mathrm{e},ij}}_{\text {(4)}} + \underbrace{\sum _{m}{\mathbf {F}}_{\mathrm{m},i }}_{\text {(5)}} + \underbrace{{\mathbf {F}}_{\mathrm{vol},i}}_{\text {(6)}} + \underbrace{\sum _{T}{\mathbf {F}}_{\mathrm{T},i}}_{\text {(7)}}+ \underbrace{{\mathbf {F}}_{\mathrm{rep},i} + {\mathbf {F}}_{\mathrm{adh},i}}_{\text {(8)}} + \underbrace{{\mathbf {F}}_{\mathrm{mig},i}}_{\text {(9)}}. \end{aligned}$$

The terms denote (1) node–node friction of nodes belonging to the same cell, to mimic damping by the CSK of two nodes move relatively to each other; (2) node–node friction of nodes belonging to different cells (alternatively, the first two terms as they have the same form could be casted into one term keeping in mind that the friction coefficients among nodes of the same cell and of a cell with another cell may differ); (3) friction between nodes of the cell and extracellular matrix (ECM) or liquid; (4) nodal forces due to CSK in-plane elasticity; (5) nodal forces due to CSK bending elasticity; (6) nodal volume force terms penalizing deviations from the cells’ intrinsic volume; (7) nodal forces due to membrane area conservation; (8) nodal contact forces consisting of a repulsive and adhesive part due to interactions with a substrate or other cells; and (9) nodal active migration forces.

Inertia terms have been neglected as the Reynolds numbers of the medium circumventing the cells are very small (Odell et al. 1981); this approximation is common for cell movement (see, e.g., Van Liedekerke et al. 2015; Drasdo et al. 2007). More specifically, the matrices ${\varvec{\Gamma }}_{\mathrm{nn}}$ and ${\varvec{\Gamma }}_{\mathrm{ns}}$ represent node–node friction and node substrate (ECM) friction, respectively. $\mathbf {v}_i$ denotes the velocity of node *i*. The first term and the second term on the rhs. represent the CSK in-plane nodal elastic forces ${\mathbf {F}}_{\mathrm{e},ij}$ and the bending force ${\mathbf {F}}_{\mathrm{m},i}$. The third term on the rhs. is a volume force ${\mathbf {F}}_{\mathrm{vol},i}$ and controls the cell compressibility. We assume that cells are compressible on longer timescales controlled by in- and outflow of water. As water transport volume flow rates are small, on short timescales cell volume can be only slightly compressed by compression of the elastic structures inside the cell such as the cytoskeleton; hence, the cell exhibits a near incompressible behavior. On longer timescales, the cell response may become more complex due to intracellular adaptations (Monnier et al. 2016). The force ${\mathbf {F}}_{\mathrm{T},i}$ accounts for resistance against isotropic expansion of the cell membrane. The two terms (${\mathbf {F}}_{\mathrm{adh},i}$, ${\mathbf {F}}_{\mathrm{rep},i}$) account for potential adhesion and repulsion forces on a local surface node, exerted by an external object such as other triangulated cells or rigid structures (see Fig. 1a). ${\mathbf {F}}_{\mathrm{mig},i}$ describes the migration forces acting on each node to result in a global movement of the cell. We now give more detail on how these forces and friction components can be calculated.

*Friction terms (1–3):* The matrices ${\varvec{\Gamma }}_{\mathrm{nn}}^{\mathrm{c}}$, ${\varvec{\Gamma }}_{\mathrm{nn}}^{\mathrm{cc}}$ and ${\varvec{\Gamma }}_{\mathrm{ns}}$ in Eq. 1 represent node–node friction and node substrate friction tensors, respectively. Friction between two nodes of the same cell mimics damping in the cortical CSK. Nodal friction terms with the cytosol are not explicitly accounted for but are partially incorporated in the node–node friction terms. Node-node friction from different cells the mimics the friction when their membranes slide along each other. Individual friction coefficients for two nodes are denoted as $\gamma _{k}$ where *k* can refers to the nature of the contact (i.e., nodes on the same cell; nodes of two different cells; friction of a node with extracellular matrix; friction of a node with a liquid). Furthermore, we distinguish between friction in parallel ($\gamma _{||}$) and normal ($\gamma _{\perp }$) direction to the relative motion. If the parallel and normal coefficients are not equal (anisotropy), the friction tensor becomes

2 $$\begin{aligned} {\varvec{\Gamma }}_{\mathrm{nn},ij} = \gamma _{\perp } (\mathbf {e}_{ij}\otimes \mathbf {e}_{ij}) + \gamma _{||} (I - \mathbf {e}_{ij}\otimes \mathbf {e}_{ij}), \end{aligned}$$

with $\mathbf {e}_{ij}=(\mathbf {r}_j-\mathbf {r}_i)/||\mathbf {r}_j-\mathbf {r}_i||$, where $r_i$, $r_j$ denote the position of the nodes of a cell. *I* is the $3\times 3$ identity matrix, while $\otimes$ denotes the dyadic product. The nodal cell–substrate friction $\varGamma _{\mathrm{ns}}$ can represent viscous resistance with liquid, ECM, capillaries or membranes. If the cell is spherical and the medium is isotropic, then $\varGamma _{\mathrm{ns}}$ is a diagonal matrix.^2^ It can be reasonable to split the friction coefficients—which have unit Ns/m—into a product of a friction coefficient per surface area (unit $\hbox {Ns}/\hbox {m}^3$) and the shared surface area associated with the interaction of the two nodes (unit $\hbox {m}^2$). The nodal areas are calculated in the contact model (See Contact force model). In case of friction of a node *i* with an external medium, this surface area is the Voronoi region area of that node (from the dual graph of the surface triangulation), which is determined by the neighboring nodes (see Van Liedekerke et al. 2017).

**Fig. 2:**
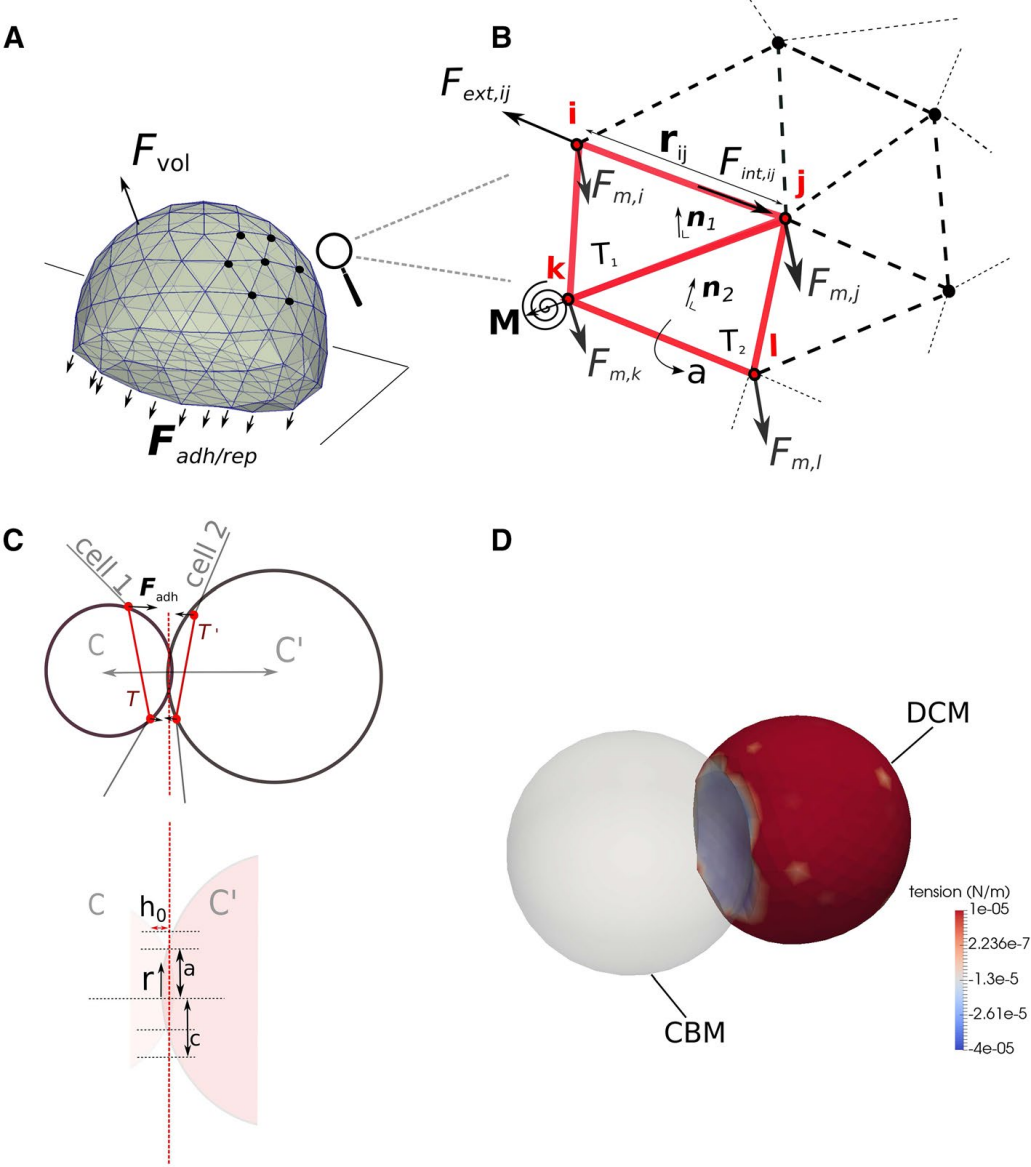
**a**, **b** Cartoon of adhering cell and detail of the force elements acting in the cell surface triangulation. **c** Projection of a triangle pair ($T,T'$) belonging to different cells to compute the cell–cell interaction force ${\mathbf {F}}_{\mathrm{adh}}$on a 2D plane for illustration. Note that in the (2D) projection, a triangle in 3D gives only two nodes, whereas the circumscribing spheres are represented as circles ($C, C'$) each with well defined radii. **d** Simulation snapshot of adhesion between a CBM and a deformable cell model (DCM). The color bar is according to membrane tension (see Eq. 8)

*Cytoskeleton in-plane elasticity—term (4) in Eq.* 1: The CSK in-plane elasticity is controlled by the viscoelastic elements. The elastic forces in the network of a cell cortex are in this model mimicked by linear springs with spring stiffness $k_{\mathrm{s}}$. In combination with the node–node friction coefficients $\gamma _{\mathrm{int}}$ which endow the terms (1) of Eq. 1, the elements become viscoelastic. Complex viscoelastic elements can be constructed by combining several springs and dashpots or using nonlinear springs.

Let us first focus on a nodal subsystem in Eq. 1 in which the spring and friction terms are schematically positioned in parallel, representing a solid like behavior so that the elements after release of an external force relax back to its original length. This is called the Kevin–Voigt Element (KVE) (Fig. 5d). Consider the internal force $F_{\mathrm{int}}$ originating from the Kelvin–Voigt viscoelastic element between node nodes *i* and *j* (for simplicity $\gamma _{||} = \gamma _{\perp }=\gamma$).

3 $$\begin{aligned} \begin{aligned} {\mathbf {F}}_{\mathrm{int},ij}&= {\mathbf {F}}_{\mathrm{e},ij} - \gamma \mathbf {v}_{ij} \\&= -\,k_{\mathrm{s}}(l_{ij}-l^0_{ij})\mathbf {e}_{ji} - \gamma \mathbf {v}_{ij}, \end{aligned} \end{aligned}$$

where $l^0_{ij}=||\mathbf {r}^0_{ij}||=||\mathbf {r}^0_{j} - \mathbf {r}^0_{i}||$ and $l_{ij}=||\mathbf {r}_{ij}||$ are the initial (cell at rest) and actual lengths between the nodes, and $\mathbf {v}_{ij}=\mathbf {v}_{i}-\mathbf {v}_{j}$ is their relative velocity. The force balance equation with external forces ${\mathbf {F}}_{\mathrm{ext}}$ (external forces can be for instance adhesion forces between nodes, see further) demands that ${\mathbf {F}}_{\mathrm{ext}}+ {\mathbf {F}}_{\mathrm{int}}=\mathbf {0}$, hence:

4 $$\begin{aligned} {\mathbf {F}}_{\mathrm{ext},ij} -k_{\mathrm{s}}(l_{ij}-l^0_{ij})\mathbf {e}_{ji} - \gamma \mathbf {v}_{ij} = \mathbf {0}. \end{aligned}$$

Contrary, a Maxwell element (ME) simulates a fluid-like extension of the cortex. In the ME, the friction element and spring element are schematically positioned in series (see Fig. 5d), which is why an external force leading to an extension of the element is damped but removal of the force does not lead to relaxation of the element back to its original length characteristic for a fluid behavior. The equation of the internal force in the element ${\mathbf {F}}_{\mathrm{int}}$ (assuming isotropic constants) reads

5 $$\begin{aligned} \frac{\dot{{\mathbf {F}}}_{\mathrm{int},ij}}{k_{\mathrm{s}}} + \frac{{\mathbf {F}}_{\mathrm{int}, ij}}{\gamma _{ME}} =\mathbf {v}_{ij}. \end{aligned}$$

This equation contains a derivative of the force, which can be approximated by $\dot{{\mathbf {F}}}_{\mathrm{int}} = ({\mathbf {F}}_{\mathrm{int}}(t)-{\mathbf {F}}_{\mathrm{int}}(t-\Delta t)) /\Delta t$. From the force balance between external and internal forces, we find now:

6 $$\begin{aligned} {\mathbf {F}}_{\mathrm{ext},ij}(t) + \frac{{\mathbf {F}}_{\mathrm{int},ij}(t-\Delta t)}{1+k_{\mathrm{s}}\Delta t /\gamma _{ME}} +\frac{k\Delta t}{1+k_{\mathrm{s}}\Delta t /\gamma _{ME}} \mathbf {v}_{ij} = \mathbf {0}. \end{aligned}$$

This equation thus involves the evaluation of the internal force on the previous time step.^3^

The linear spring constant $k_s$ for a sixfold symmetric triangulated lattice can be related approximately to the cortex Young modulus $E_{\mathrm{cor}}$ with thickness $h_{\mathrm{cor}}$ by Boal (2012)

7 $$\begin{aligned} k_{\mathrm{s}}\approx \frac{2}{\sqrt{3}}E_{\mathrm{cor}}h_{\mathrm{cor}}. \end{aligned}$$

Furthermore, the total elastic in-plane CSK forces can be related to a local in-plane stress using the virial formula:

8 $$\begin{aligned} \sigma _i =\frac{\sqrt{3} }{N_i}\sum _{j \in N_i} \frac{F_{\mathrm{int}, ij}}{l_{ij}}, \end{aligned}$$

where $N_i$ is the coordination number of node *i*, $F_{\mathrm{int}, ij}$ is the total elastic force between *i* and *j*.

We here do not assume an active contractile state of the cell. Cell contractility could be included in the model as an extra active elastic force term by, e.g., changing the element rest length $l^0$ (see Odell et al. 1981), but this is not considered in this paper.

*CSK bending force—term (5) in Eq.* 1: The bending resistance from the cortex is incorporated by the rotational resistance of the hinges determined by two adjacent triangles $T_1= \{ ijk \}$ and $T_2 = \{ ijl \}$ (see Fig. 2b). This permits to define the bending moment *M*:

9 $$\begin{aligned} M= k_{\mathrm{b}}\sin (\theta - \theta _{0}) \end{aligned}$$

where $k_{\mathrm{b}}$ is the bending constant specified below and $\theta$ is the angle between the  normal vectors to the triangles $\mathbf {n}_{1 },\mathbf {n}_{2 }$ , determined by their scalar product $(\mathbf {n}_{1 } \mathbf {n}_{2 })=\cos (\theta )$. $\theta _0$ is the angle of spontaneous curvature. The moment *M* can be transformed to an equivalent force system ${\mathbf {F}}_{\mathrm{m},z}$ ($z \in \{ ijkl \}$) for the triangles $T_1$ and $T_2$ where for $T_1$ we can compute ${\mathbf {F}}_{\mathrm{m},i} = M / l_{1} \mathbf {n}_1$ using $l_1$ as the distance between the hinge of the triangle pair and the point *i*, and similar expression for ${\mathbf {F}}_{\mathrm{m},l}$. The forces working on nodes *j*, *k* must fulfill ${\mathbf {F}}_{\mathrm{m},i}+{\mathbf {F}}_{\mathrm{m},j}+{\mathbf {F}}_{\mathrm{m},k}+{\mathbf {F}}_{\mathrm{m},l}=0$ to conserve momentum. The bending stiffness of the cortex is approximated by

10 $$\begin{aligned} k_{\mathrm{b}}\approx \frac{E_{\mathrm{cor}}h^3}{12(1-\nu ^2)}, \end{aligned}$$

where $\nu$ is the Poisson’s ratio of the cortex.

*Volume force—term (6) in Eq.* 1:

The nodal contact forces with the cytosol are in the standard model accounted for by the volume forces. These regulate the volume changes according to the applied pressure and the bulk modulus property $K_{\mathrm{V}}$ of the cell. The compressibility of the cell depends on volume fraction of water in the cytosol, the CSK volume fraction and structure, and the compressibility of the organelles. In addition, it may be influenced by the permeability of the plasma membrane for water, the presence of caveolae (Sinha et al. 2011), and active responses in the cell. We calculate the internal pressure in a cell by the logarithmic strain for volume change:

11 $$\begin{aligned} p = -\,K_{\mathrm{V}}\log \left( \frac{V}{V_0}\right) , \end{aligned}$$

whereby *V* is the actual volume and $V_0$ is the reference volume, i.e., the volume of the cell not subject to compression forces. For small deviations of *V* from $V_0$, $p \approx K_{\mathrm{V}}(V-V_{0})/V_{0}$. Within our model, the volume *V* of the cell is computed summing up the volumes of the individual tetrahedra that build up the cell. The nodal force is obtained by multiplying the pressure with the nodal Voronoi region area $S_i$ (see Van Liedekerke et al. 2017), i.e., ${\mathbf {F}}_{\mathrm{vol},i}=pS_i{\mathbf {R}}^n$ where ${\mathbf {R}}^n$  is the normalized curvature vector computed for that node (see contact force model).

Equation 11 expresses isotropic compression only. As $K_{\mathrm{V}}\gg E_{\mathrm{cor}}h_{\mathrm{cor}}/R_{c}$ (see Tinevez et al. 2009), $K_{\mathrm{V}}$ controls the overall compressibility of the cell while the mechanical stiffness of the cortex plays a minor role herein.

In case the internal CSK would be explicitly represented by internal structural elements not considered in the simulations of this work, those elements would contribute to both volume compression and shear forces.

*The membrane area conservation force—term (7) in Eq.* 1: A lipid bilayer membrane resists to expansion of its area, but only little to shear forces. This can be expressed by the force magnitude:

12 $$\begin{aligned} {F}_{\mathrm{T}} = k_{\mathrm{mem}}(A-A_{0})/A_0. \end{aligned}$$

Here $k_{\mathrm{mem}}$ is the area compression stiffness and $A_0$, *A* are the reference and the current surface areas of the cell, respectively. These can be obtained by summing all the individual triangle areas, i.e., $A=\sum _k{a_k}$ of the cell surface, where $a_k$ is the surface area of a triangle $T_k$ (see Fig. 2b). Note that $A_0$ is not necessarily constant as the cell can grow. The direction of the force ${\mathbf {F}}_{\mathrm{T}_k}$ is from the barycenter of each triangle’s plane outwards. The parameter $k_{\mathrm{mem}}$ for area conservation forces in the membrane is obtained by rescaling the area compression modulus $K_{\mathrm{A}}$ with the vertex resolution l^0^:

13 $$\begin{aligned} k_{\mathrm{mem}}\approx K_{\mathrm{A}}l^{0}. \end{aligned}$$

*Cell–cell contact model forces—terms (8) in Eq.* 1: Whereas in a CBM, cells interact through central forces described by (modified) Hertz or JKR theory for adhesive spheres, in DCM the interaction forces need to be defined for each node individually, thereby endowing representation of local surface heterogeneities. This can be achieved by pairwise potential functions (Van der Waals, Morse) between nodes which mimic the effect of short-range repulsion and long-ranged attraction forces of molecules (Tamura et al. 2004; Rejniak and Anderson 2008; Sandersius and Newman 2008; Jamali et al. 2010). While straightforward to implement, this approach poses some problems with respect to the scalability and calibration of the parameters for these potentials. Furthermore, pairwise potentials are not efficient in avoiding unwanted interpenetration of two approaching cells. The alternative approach followed in this work (which largely avoids these problems) is based on Maugis–Dugdale theory. This has been successfully applied to predict red blood cell spreading dynamics on surfaces (Odenthal et al. 2013). The Maugis–Dugdale theory for adhering bodies is a generalization of the JKR theory for spheres (Maugis 1992). It captures the full range between the Derjaguin–Muller–Toporov (DMT) zone of long reaching adhesive forces of a soft homogeneous isotropic elastic sphere and small adhesive deformations of the Johnson–Kendall–Roberts (JKR) limit of a hard homogeneous isotropic elastic sphere of short interaction ranges. Here, we assign each triangle of the cell surface with a circumscribing sphere reflecting the local curvature. Two triangles belonging to different cells can interact by collision of their assigned spheres. To compute the magnitude of these interactions, we use Maugis–Dugdale stress formula.

In our model, Maugis–Dugdale theory is applied to a discrete system of triangles, which constitute the cell surface. This assumes a quasi-continuous distribution of cadherin bonds at the cell contact area.^4^ Because the cell curvature is not constant as it would be for a perfect sphere, we locally estimate the curvature from the triangulated structure (see Fig. 2c) using the discretized Laplace–Beltrami operator, which is an approximation function to associate a mean curvature vector to a discretized surface or boundary curve as an alternative to the approximation by the angle between the surface normal vectors (Drasdo and Forgacs 2000). In this way every triangle of the cell can be associated with a local curvature vector ${\mathbf {R}_i}$ for which a circumscribing sphere can be defined. An interaction between two cells defines several pairwise triangle–-triangle interactions ($T,T'$). One pair of triangles defines a pair of circumscribing spheres ($C,C'$) with radii determined by the curvatures, and a common contact plane to which their triangles are projected (Fig. 2c, dashed red line).

The local stress at the contact, which depends on the radius of both spheres and the adhesion energy can then be computed using the adhesive Maugis–Dugdale stress component:

14 $$\begin{aligned} p_a = {\left\{ \begin{array}{ll} -\frac{\sigma _0}{\pi } \arccos \left( \frac{2a^2-c^2-r^2}{c^2-r^2}\right) &{} \text{ if } 0\le {r}\le a \\ -\sigma _0 &{} \text{ if } a\le {r}\le 0. \end{array}\right. } \end{aligned}$$

In this formula, *r* is the distance from the contact point, *a* is the effective contact radius (pure Hertz contact) and *c* is the radius of the adhesive zone (see Fig. 2, bottom). We then compute the Tabor coefficient (Johnson and Greenwood 1997):

15 $$\begin{aligned} \lambda = \sigma _0\left( \frac{9\hat{R}}{2\pi W\hat{E^2}} \right) ^{1/3}, \end{aligned}$$

where $\hat{E}$ and $\hat{R}$ are the reduced elastic moduli and radius of the two objects in contact.^5^ The tension $\sigma _0$ is the maximum adhesive tension from a Lennard-Jones potential and is related to the specific adhesion energy *W* by $W=h_0\sigma _0$. Here $h_0$ is the typical effective adhesive range that reflects the attractive cutoff distance between the bodies. We set $h_0 = 2\times 10^{-8}{m}$ in all the simulations (Leckband and Israelachvili 2001; Odenthal et al. 2013). After this, *c* can be computed from $m=c/a$ for which holds:

16 $$\begin{aligned}&\frac{\lambda }{2}\left( \frac{4a^3 \hat{E}}{3\pi W\hat{R}^2}\right) ^{2/3}\left[ (m^2-2)\sec ^{-1}m +\sqrt{m^2-1}\right] \nonumber \\&\quad + \frac{4 \lambda ^2}{3}\left( \frac{4a^3 \hat{E}}{3\pi W\hat{R}^2}\right) ^{1/3} \left[ (m^2-2)\sec ^{-1}m -m +1\right] =1. \end{aligned}$$

On the other hand, the repulsive part is given by the Hertz pressure:

17 $$\begin{aligned} p_r=\frac{2\hat{E}}{\pi \hat{R}}\root \of {a^2-c^2}. \end{aligned}$$

The total stress distribution between the two spheres is thus $p_a+p_r$. The total interaction force ${\mathbf{F}}_{\mathrm{adh},i} + {\mathbf{F}}_{\mathrm{rep},i}$ between a pair of triangles is obtained by integrating this stress using standard Gauss quadrature rules over the surface area common of the two triangles that have been projected on the contact plane. Once the force is known, it is distributed onto the nodes of both triangles, with the total force on the first triangle opposite in sign to the one of the second, to conserve total momentum. Note that for each triangle which has a contact area *A* with another triangle, each node associated with this triangle acquires a contact area *A* / 3. For more details about the implementation, see Odenthal et al. (2013) and Smeets et al. (2014, 2019).

Importantly, this interaction model also permits to simulate interactions between a triangulated body and a smooth surface such as spheres or planes having fixed curvature. In such case, the contact establishes simply between the sphere assigned to a triangle, and the sphere that represents the object as a whole. This allows to implement a relatively simple algorithm that defines the handshake between a DCM and a CBM (see example Fig. 2d).

*Cell migration force—term (9) in Eq.* 1: The migration force ${\mathbf {F}}^{\mathrm{mig}}$ is usually an active force, representing the random micro-motility of a cell. Migration of cells involves complex mechanisms such as filopodia formation and cell contractility, and may be modeled as such (see, e.g., Tozluoğlu et al. 2013; Kim et al. 2018), but for the sake of simplicity we do not resolve the migration in such detail and instead lump the different mechanisms into one net force, which is homogeneously distributed to the nodes the cell. For specific applications, the forces might be in-homogeneously distributed. In the absence of influences that impose a certain direction or persistence, it is commonly assumed that the migration force is stochastic, formally resulting in ${\mathbf {F}}^{\mathrm{mig}}={\mathbf {F}}^{\mathrm{ran}}$, with $\langle {\mathbf {F}}^{\mathrm{ran}}\rangle =\mathbf {0}$, and $\langle {\mathbf {F}}^{\mathrm{ran}}(t)\otimes {\mathbf {F}}^{\mathrm{ran}}(t')\rangle = {\mathbf {M}} \delta (t-t')$, where ${\mathbf {M}}$ is an amplitude $3 \times 3$ matrix and relates to the diffusion tensor ${\mathbf {D}}$ of the cell. As cell migration is active, depending on the local matrix density and orientation of matrix fibers, the autocorrelation amplitude matrix ${\mathbf {M}}$ can a priori not be assumed to follow a fluctuation–dissipation (FD) theorem. However, “measuring” the position of a cell in the simulations the position autocorrelation function might be experimentally used to determine the diffusion tensor using $\langle ((\mathbf {r}(t+\tau )-\mathbf {r}(t))\otimes (\mathbf {r}(t+\tau )-\mathbf {r}(t))\rangle = 6{\mathbf {D}}\tau$, and ${\mathbf {M}}$ be calibrated such that the numerical solution of the equation of motion for the cell position reproduces the experimental result for the position autocorrelation function. For example, in a homogeneous environment ${\mathbf {M}}$ can be casted into a form formally equivalent to the FD theorem, leading to a $k_{\mathrm{B}}T$-equivalent for cellular systems, that is controlled by the cell itself (Van Liedekerke et al. 2015; Drasdo and Hoehme 2012).

On the other hand, if cells migrate in response to a morphogen gradient, an additional directed force ${\mathbf {F}}^{\mathrm{mor}}$ into the direction of the concentration gradient of the morphogen may occur. The total migration force for the cell reads then ${\mathbf {F}}^{\mathrm{mig}}= {\mathbf {F}}^{\mathrm{ran}}+{\mathbf {F}}^{\mathrm{mor}}$. In the liver lobule example simulations (Model III in Sect. 3.3), we assume that only “leader” cells have the capability of directed migration. These are the cells in the lobule that are located at the interface to the pericentral necrotic lesion.

Note finally, we assume here momentum transfer to the ECM by the ECM friction and active micro-motility term but we do not model the ECM explicitly.

#### Cell growth and mitosis

During progression in the cycle, a cell grows by acquiring dry mass and water, eventually doubling its volume. This volume growth in both, interphase consisting of the $G_1$, *S* and $G_2$ phase, and mitosis phase may be described by an increase in the radius of the cell. We assume here that for a cell in free suspension during growth stage, the reference volume of the cell $V_0$ gradually increases and is updated according to:

18 $$\begin{aligned} V_{0}(t+\Delta t)= V_{0}(t)+\alpha \Delta t, \end{aligned}$$

where $\alpha$ is the growth rate for simplicity assumed to be constant during the cycle, which can be justified if we look at timescales much larger than one cell division (Van Liedekerke et al. 2019). $\alpha$ is chosen such that the volume of a cell doubles in the experimentally observed cell cycle duration.

Cell growth and division in the CBM (see, e.g., Drasdo et al. 2007) is straightforward to implement, but involved for the DCM, requiring a multistep procedure.

**Fig. 3:**
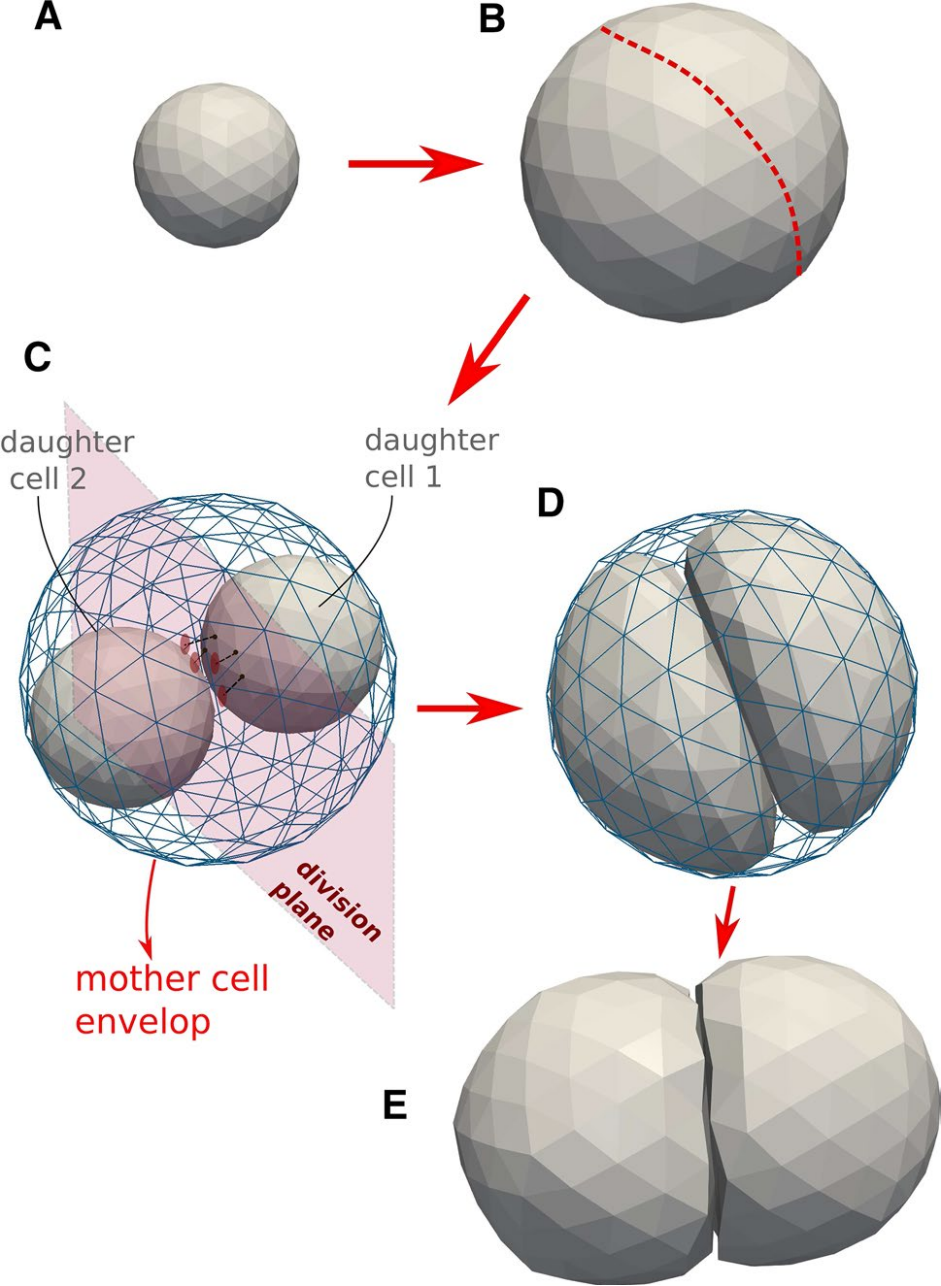
Model algorithm of the cell cycle of deformable cells: **a**, **b** cell growth (double volume by increasing the radius). **b** Choose division direction randomly (assign nodes to one side). **c** Add two default cells as daughter cells to center of masses. Project nodes of the daughter cells to surface of division plane if they intersect it. A sub-simulation is run to come rapidly to situation D. **d** After the sub-simulation, mother envelope is removed. **e** Division stage finished: two mechanically relaxed daughter cells

*Cell growth*: During cell growth, the volume of the cell is increasing (Fig. 3a, b). We model this by an update of the reference volume $V_{0}$ following Eq. 11, update of the reference values of the cell surface triangle areas, and update of the spring lengths. The reference triangle area $A_0$ for each triangle, and spring rest length $l^0$ for each viscoelastic element are recomputed every time step $\Delta t$ according to $A_0(V_{0}(t+\Delta t)/V_{0})^{2/3}$ and $l_0(V_{0}(t+\Delta t)/V_{0})^{1/3}$, respectively. The coordinated update of $V_0$, $A_0$ and $l^0$ in the algorithmic implementation of the model is necessary to ensure that no additional volume penalty forces or other artifacts are generated during growth. Note that at this moment, growth inhibition due to excessive external mechanical stress (e.g., Drasdo and Hoehme 2012) can be easily included in our model, but in the scope of this paper we do not consider it further.

*Cell death*: When a cell dies, it can be either removed instantly from the simulation, or gradually shrink (lysis). Algorithmwise, lysis can be regarded as the inverse of the growth process. However, during lysis the mechanical parameters of the cell may change. The process may continue until a certain cell volume has been reached, below which the cell cannot longer shrink. Finally, a lysed cell may be removed completely from the simulation (e.g., by phagocytosis).

*Cell division*: During cytokinesis, the continuous shrinking of the contractile ring, together with the separation of the mitotic spindle, gradually creates the new daughter cells. After mitosis the cell has split up in two adhering daughter cells. An analytical approach describing this process can be found in Turlier et al. (2014). An approach with 2D deformable cells has been previously proposed (e.g., Jamali et al. 2010). In a first attempt, we had implemented such an algorithm in 3D but this turned out to be prone to numerical instabilities, as triangles on the side of the contractile ring tended to be extremely stretched while nodes accumulated at the position of the contractile ring. Hence, simulations of these processes in DCM in 3D would require a complex re-meshing process for the surface to leave the local stresses unchanged but avoiding numerical instabilities. An implementation of these steps turned out to be not only challenging, but also computationally time consuming.

As we are merely interested in long-term effects (i.e., several hundreds of cell divisions), and as the cytokinesis is a short process compared to the duration of the entire cell cycle, we avoid these particular tedious intermediate steps in our model, and instead directly create two new adhering cells that are enclosed by the mother cell just before its division as pictured in Fig. 3.

First, a division plane is chosen, which determines the direction into which the cells divide. The label of the division plane on the surface of the mother cell can be associated with the position of the contractile ring. The orientation of this plane may be chosen randomly or into a preferred direction, and splits the mother cells into two halves each bounded by part of the surface of the mother cell and the division plane (see Fig.3b, c); note that the mother cell in the figure is spherically shaped, but the algorithm works for arbitrary cell shapes as well). Then, the centers of the two future daughter cells on both sides of the plane are computed as the two centers of mass of the nodes that form each of the two halves, and each of those two centers of mass is associated with the center of a new spherically shaped daughter cell (Fig. 3c). The radii of the daughter cells are chosen such that they are both contained by the mother cell. To ensure the daughter cells are not overlapping at this stage, those nodes that would overlap with the division plane are projected back on the division plane. Each of the daughter cells has now as border approximately a half of the mother cell envelop and the division plane that it shares with the other daughter cell. At this stage however, the radii (and volumes) of the daughter cells are not yet those they each should have, i.e., half of the volume of the mother cell.

To achieve this, a *sub-simulation*^6^ is performed where the cell volume and strut lengths of the viscoelastic network are reset to their reference values for a cell half the size of the mother cell. During the sub-simulation, we artificially set all friction coefficients of the daughter cells to very small values. This “inflates” the two daughter cells rapidly. On the other hand, we momentarily freeze the positions of the nodes of the mother cell. As a consequence, the two cells will rapidly adapt their shapes to the limiting shape of the mother cell “cocoon” and the division plane. The repelling interactions between the triangulated envelop of the mother cell and daughter cells ensure that the latter stay inside. We thus arrive in this step at a system of three triangulated cells: one fixed mother cell, and two encapsulated daughter cells with forming a shape approximately that of mother cell just before cell division.

After the sub-simulation, all the friction coefficients are reset to their normal values, the division plane is discarded and the daughter cells start adhering to each other because the mutual adhesion forces are invoked. The mother cell envelop is removed from the simulation and the daughter cells interact again with their environment. The system can relax slowly toward a mechanical equilibrium (Fig. 3d) over a time span equal to the mitosis phase duration.

### Center-based model (CBM)

Center-based models (CBM) are well-established modeling approaches where cells have the same features as in DCM, but the cell shape is approximated as a simple geometrical object. The cell shape is not explicitly modeled but only captured in a statistical sense. The cells are represented by homogeneous elastic and adhesive spheres (see Fig. 1a, bottom), and interact through pairwise forces (e.g., Hertz, JKR), which are computed from a virtual overlap of both cell geometries. The equation of motion is similar to that of DCMs, yet the forces are here directly applied to the cell centers (for details, see Sect. 2.3). During division, two new cells are created next to each other that replace the mother cell. In our work, we will use the CBM for comparative runs in the simulations for liver lobule regeneration (Hoehme et al. 2010), see Sect. 3.3.

### Forces, equations of motion and cell division

We here only briefly recapitulate the basic components of CBM: for more detailed information, we refer to literature (see, e.g., Drasdo and Höhme 2005; Drasdo and Hoehme 2012; Van Liedekerke et al. 2015). In CBM, cells are usually represented as spheres. The equation of motion for a cell *i* reads:

19 $$\begin{aligned} {\varvec{\Gamma }}^{\mathrm{cs}}_{i} \mathbf {v}_i + \sum _{j}{\varvec{\Gamma }}^{\mathrm{cc}}_{ij} (\mathbf {v}_i-\mathbf {v}_j) = \sum _{j}{\mathbf {F}}^{\mathrm{int}}_{ij} + {\mathbf {F}}^{\mathrm{sub}}_{i} + {\mathbf {F}}^{\mathrm{mig}}_{i} \end{aligned}$$

For the same reasons as for the DCM, inertia is neglected as cell movement occurs. The first term on the lhs. denotes the friction of cells with the substrate or extracellular matrix, the second term cell–cell and—in simulations of the regenerating liver lobule as explained in the introduction of this paper—cell-sinusoid friction forces. Sinusoids are modeled in this work as immobile chain of slightly deformable spheres (hence $\mathbf {v}_j=0$ for sinusoidal elements *j*) with the radius equal to the sinusoidal radius found experimentally (Hammad et al. 2014). For cell–cell friction, the velocities $\mathbf {v}_j$ will generally be not zero. ${\mathbf {F}}^{\mathrm{int}}_{ij}$ denotes interaction forces on cell *i* from repulsion or adhesion with other cells *j* or static blood vessel cells (see Sect. 3.3). The force ${\mathbf {F}}^{\mathrm{sub}}_{i}$ reflects adhesive/repulsive interactions with a flat substrate or wall. ${\mathbf {F}}^{\mathrm{mig}}_i$ denotes the total migration force which has a random part and may also have a directed term (see explanation DCM). The friction terms involve tensors for the cell–cell friction (${\varvec{\Gamma }}^{\mathrm{cc}}_{ij}$) and cell–substrate friction (${\varvec{\Gamma }}^{\mathrm{cs}}_i$). The friction tensors are computed in the same way as for the DCM, with the nodes of the DCM being replaced by the actual cell centers in the CBM (Drasdo and Hoehme 2012).

The cells in CBM interact by pairwise forces having a repulsive and adhesive part, which are characterized by a function of the geometrical overlap $\delta _{ij} = R_i +Rj - d_{ij}$. As in Chu et al. (2005), we assume here that cell adhesion forces can be described by Johnson–Kendal–Roberts (JKR) model, approximating cells by isotropic homogeneous sticky elastic bodies that are moderately deformed if pressed against each other. The interaction force is computed by

20 $$\begin{aligned} F^{\mathrm{int}}_{ij}= \frac{4 \hat{E}}{3 \hat{R}} \left[ a(\delta _{ij})\right] ^3 - \sqrt{ 8 \pi W \hat{E} \left[ a(\delta {ij})\right] ^3}. \end{aligned}$$

The contact radius *a* in Eq. 20 allows to compute the cell–cell contact area, and can be obtained from Pathmanathan et al. (2009):

21 $$\begin{aligned} \delta _{ij} = \frac{a^2}{\hat{R}} - \sqrt{\frac{2 \pi W }{\hat{E}} a}. \end{aligned}$$

In the latter equations, $\hat{E}$ and $\hat{R}$ are defined as

$$\begin{aligned} \hat{E} = {\left( \frac{1-\nu _i^2}{E_i} + \frac{1-\nu _j^2}{E_j} \right) }^{-1} \; \text {and} \; \hat{R} = {\left( \frac{1}{R_i} + \frac{1}{R_j} \right) }^{-1}, \end{aligned}$$

with $E_i$ and $E_j$ being the Young’s moduli, $\nu _i$ and $\nu _j$ the Poisson numbers and $R_i$ and $R_j$ the radii of the cells *i* and *j*, respectively. W = ρ_m_ W_b_ is the specific surface adhesion energy obtained by multiplying the surface density of adhesion molecules ρ_m_ with the adhesion energy W_b_ stored in a single adhesive bond. Note that the Young moduli in the center-based model should be chosen such that they correspond as much as possible to the elastic properties of the DCM. In particular, we warrant here that for a CBM’s Young modulus and Poisson’s ratio, $E=K_{\mathrm{V}}/3(1-2\nu )$ where $K_{\mathrm{V}}$ is the compression modulus of the DCM cell (Sect. 2.1). Note that for a CBM, the elastic properties of the cortex and membrane cannot be identified a priori.

During the cycle the intrinsic volume of a mother cell doubles before it splits into two daughter cells. The intrinsic volume is defined by the volume of the cell if it would be undeformed and uncompressed. Its true volume in case it interacts with other cells or structures cannot be exactly tracked as the CBM does mostly not permit to calculate the volume of cells interacting with other cells. Like in the DCM, we are assuming constant growth rate during the cell cycle and the intrinsic volume $V_{i}$ of cell *i* is updated every time step $\Delta t$ as in Eq. 18.

If the cell passed a critical volume $V_{\mathrm{crit}}$, the cell undergoes mitosis and two new cells with volume $V_{\mathrm{crit}}/2$ are created. In case the cell grows to twice its original value to divide as considered throughout this work, *V*_crit_ = 2V_0_. A simple version of the division algorithm consists of placing directly two smaller daughter cells in the space originally filled by the mother cell at the end of the interphase (Schaller and Meyer-Hermann 2005; Galle et al. 2005). When the two daughter cells are created, the JKR interaction force will push away the two daughter cells until mechanical equilibrium is reached. If the space filled by the mother cell is small, which is often the case for cells in the interior of a cell population, the local interaction forces occurring after replacing the mother cell by two spherical daughters can adopt large (unphysiological) values leading to unrealistic large cell displacements. This might be circumvented by intermediately reducing the forces between the daughter cells (see below). Alternatively, cells could in small steps be deformed during cytokinesis into dumbbells before splitting (Hoehme et al. 2010). In this work, we pursue the simpler approach as it resembles the cell division algorithm we use for the DCM.

#### Corrections to JKR contact forces

As mentioned above, center-based models suffer from a number of major artefacts, often ignored in simulations.

The most striking is that common pairwise contact forces (type Hertz, JKR, etc.) and contact areas become largely inaccurate when cells are densely packed and become jammed and compressed. This problem discussed in Van Liedekerke et al. (2015); Van Liedekerke et al. (2019) is comparable to the packing problem in liquid foams and emulsions as described in Höhler and Weaire (2018), Höhler and Cohen-Addad (2017). In densely packed cell aggregates, the deformation a cell, say cell *i*, experiences as a consequence of interaction with one of its neighbors, say cell *j* can be so large, that it affects the interaction cell *i* has with another neighbor cell, say cell $k\ne j$. In that case the approximation of considering the interaction forces between the pair cell *i* and cell *j* as independent of the interaction force between the pair cell *i* and cell *j* becomes inaccurate. As a consequence, even an incompressible cell characterized by Poisson ratio $\nu = 0.5$ in the JKR force model (Eq. 20) will be compressed if surrounding cells are pushed toward it. In such a situation, cell volume and cell–cell contact area are only poorly approximated by the JKR force model.

Voronoi tessellation of the positions of the cells can estimate the individual cell volumes (and hence predict realistic pressure forces) (Bock et al. 2010; Schaller and Meyer-Hermann 2005). However, in the attempt to correct the unrealistic contact forces in the center-based model upon large compression forces, we here propose a simple calibration step in which we use DCM simulations to estimate contact forces between cells. The DCM does not have the aforementioned problem because the shape and cell volume are determined at high spatial and temporal resolution. As such, there is no notion of geometrical overlap in DCM cells. A small clump of DCM cells is compressed quasi-statically while monitoring the contact forces as function of the distances between the cell centers (see “Appendix 1”, Fig. 12b). During compression, a stiffening of the contacts can be clearly observed. Performing an equivalent experiment with CBM using the JKR law results in a significant underestimation of the contact forces (Fig. 12b). To correct these, we partially follow the procedure as outlined in Van Liedekerke et al. (2019). As in this work, we keep the original JKR contact law form but modify the apparent modulus $E_{i}\rightarrow \tilde{E}_{i}$ of the cell as the cells approach each other, by gradually increasing it in Eq. 20 as the cells get more packed. The challenge lies in determining $\tilde{E}_{i}$ as a function of the compactness of the spheroid. We try to estimate the degree of packing around one cell by using the distances between that cell and its neighbors, introducing a function that depends on the *local* average of the distances $\tilde{d}_i = \sum _j \tilde{d}_{ij} /N$ (with $\tilde{d}_{ij} = 1 - d_{ij}/(R_i+R_j)$ and *N* is the total number of contacts^7^) to each of the contacting cells *j*, noticing that the cell–cell distances differ only very slightly. We here considered the case where all cells all have the same elastic modulus; for interacting cells of different parameters, the procedure might have to be adapted. Different to Van Liedekerke et al. (2019) we do not take into account the effective volume reduction of the CBM cells, because we assume that cells are not as heavily compressed. The simulated force curves of the DCM could well be reproduced with the CBM for the following simple polynomial function of 4th order:

22 $$\begin{aligned} \tilde{E}_{i}(\tilde{d}_{i})=a_{0} + a_{1}\tilde{d}^4_{i}, \end{aligned}$$

in where $a_{0} = E_{i}$ and, $a_{1}$ is a fitting constant. The best fit to the DCM contact force is shown in Fig. 12a, with $a_{1}= 4\times 10^6$. However, we note that the contact stiffness generally depends on the number of neighbors a cell has. In this experiment, every cell had initially 8 contacting neighbors, yet this number evolves to approximately 12 as the compression progresses, which is what would be expected from the Euler theorem for volume tessellation.

A side effect of this calibration method is that high repulsive forces may arise during cell division, when two new cells are created and positioned close to each other. To limit these effects, we apply the above formula in a such way, that the contact stiffness becomes gradually larger over a time period just after division, which we choose about $\sim 1/10$ of the cell cycle (roughly the duration of mitosis phase; however, smaller durations would also be possible), reaching the maximal value after this period. This is similar as what has been used in Galle et al. (2005) to avoid repulsion force cues, and ensures that cells will not separate abruptly during division. The period gives time to the cells to evolve to a local mechanical equilibrium, and can be seen as the analog of the relaxation period in the division algorithm in the deformable cell model (see Sect. 2.1.2). We have verified that small variations on this relaxation period did not influence the simulations results significantly.

## Results

### Single cell experiments

#### Calibration of DCM parameters from optical stretcher experiments

**Fig. 4:**
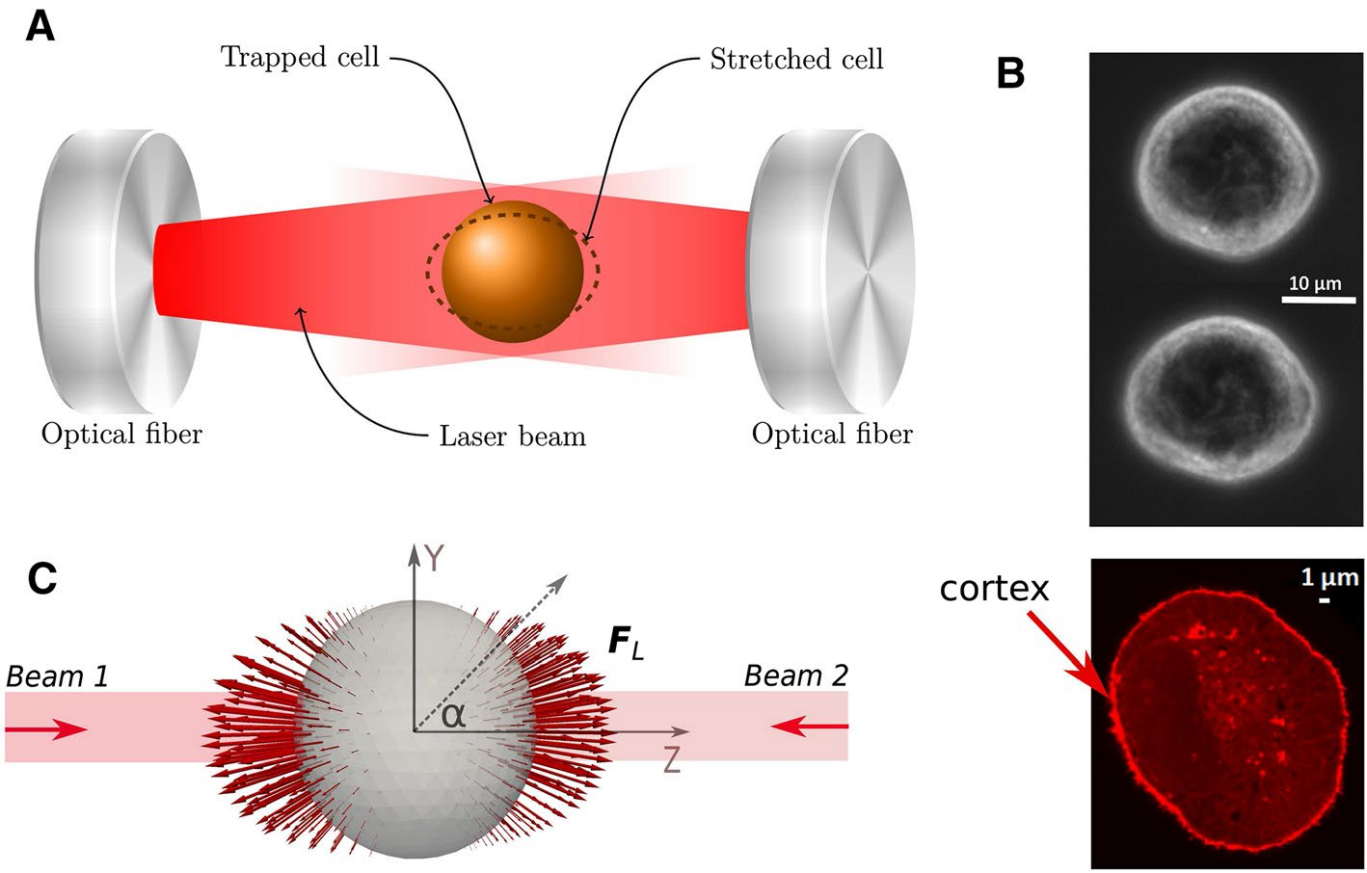
**a** Sketch of the experimental setup with a trapped cell in an optical stretcher. **b** Top: image of unstretched and stretched MDA-MB-231 cell. Bottom: actin-stained image of a MDA-MB-231 cell indicating cell cortex actin cytoskeleton. **c** Surface forces profile due to laser is visualized (red arrows) on a triangulated surface of the deformable cell. The *Z*-axis (long axis) is aligned with the laser beam, whereas the *X*- and the *Y*-axis give the direction of the short axes during deformation. ${\mathbf {F}}_{\mathrm{L}}$ is the nodal force induced by the laser power

Classical experiments such as optical stretchers, optical tweezers, and micro-pipetting techniques are used to observe the mechanical behavior of individual cells and can be used to estimate the physical range of the DCM viscoelastic network model parameters (see, e.g., Odenthal et al. 2013; Guyot et al. 2016; Fedosov et al. 2011). Here, we choose the optical stretcher experiment where, as the experiment is performed in suspension, represents a type of ground state and thus an excellent situation for the calibration of the mechanical parameters. Therefore, the optical stretcher experiment is mimicked in silico with a DCM. In the optical stretcher, two laser beams in opposite direction and faced to each other trap a cell in suspension (see Fig. 4a). The diffracted laser beams exert a surface force on the cell’s membrane and cortex, deforming it toward the beam direction. Increasing the laser power yields a higher optical stretching force. At the same time, the deformation along two perpendicular directions is measured using image analysis, yielding information on cell shape. We refer to Guck et al. (2001, 2005) and Grosser et al. (2015) for more details of the setup and conditions in such an experiment.

New data of optical stretcher experiment were generated using MDA-MB-231 breast cancer cells. These cells had an average radius of $8.8 \pm 1.3\,{\upmu \mathrm{m}}$ ($N= 100$ samples). Actin staining further revealed average approximate cortex thickness of $h_{\mathrm{cor}} \sim 500{\mathrm{nm}}$ (Fig. 4b).

**Fig. 5:**
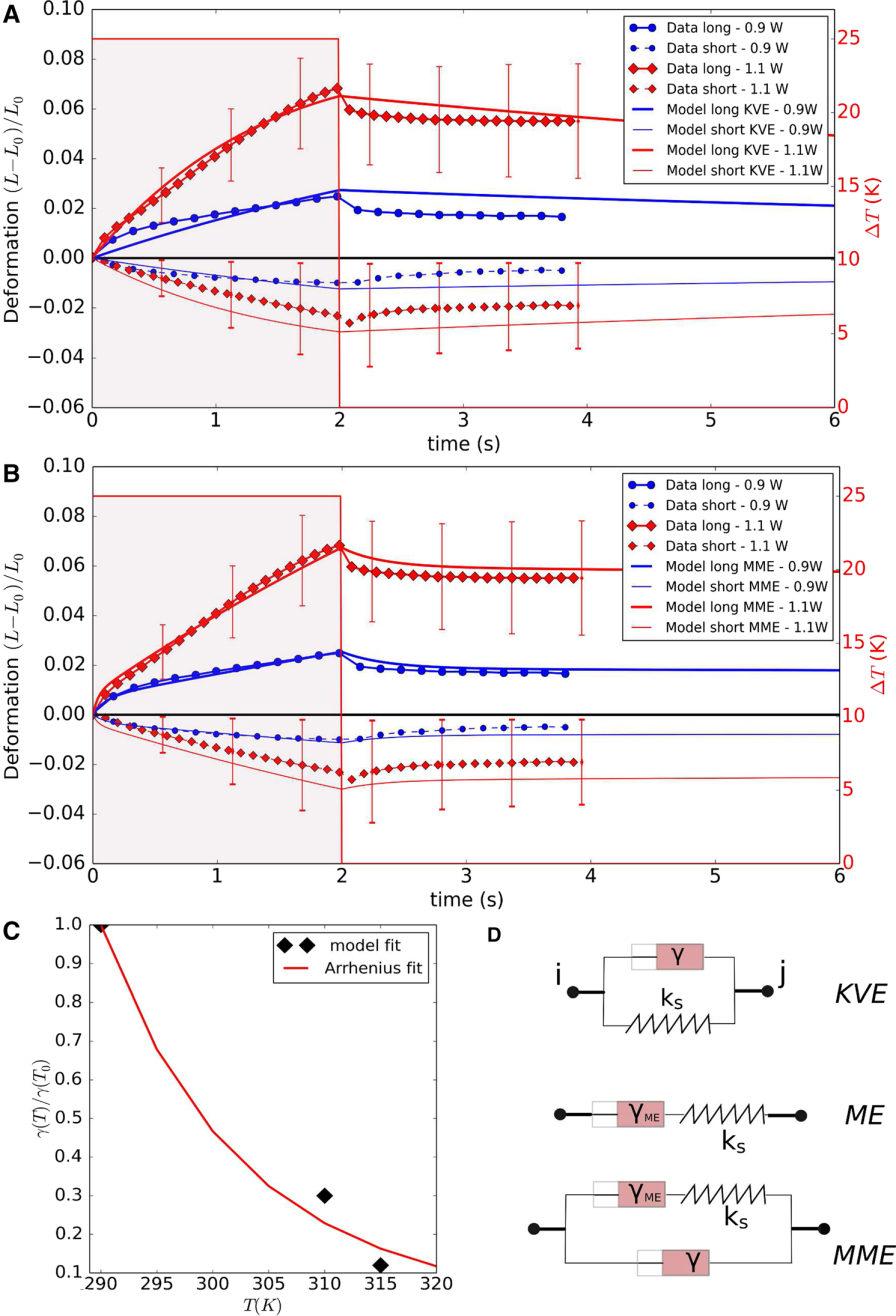
**a**, **b** Comparison of the simulated MDA-MB-231 cell deformation with the experimental data in an optical stretcher, using laser powers of $900\,{\mathrm{mW}}$ and $1100\,{\mathrm{mW}}$. **a** The model was first fitted using a Kelvin–Voigt model (KVE) and assuming temperature dependent friction coefficients for the cytoskeleton. **b** The model then was fitted assuming a modified Maxwell model (MME) but using the same temperature dependence as for KVE. For sake of clarity, the error bars are only shown for some data points, and only for the $P_0=1100\,{\mathrm{mW}}$ case. The errors bars on the data are quite large, indicating a high biological variability. **c** Temperature dependence of damping coefficients, using model fit for DCM, compared to Arrhenius’ law using an activation energy close to the value reported in Kießling et al. (2013). **d** Schematic representation of nodal force elements with springs and dampers. KVE, Kelvin–Voigt element; ME, Maxwell element; MME, modified Maxwell element

In each experiment, the laser beams were applied during a time interval of a few seconds, in which the cell continues to deform. Thereafter, the cell relaxation behavior period was monitored for several seconds. The long axis (*Z*) and short axis (*Y*) lengths changes over time are derived from analyzing the images (see data Fig. 5; the *X*-axis and *Y*-axis are by rotation symmetry with regard to the *Z*-axis assumed to be equally long). Two laser powers were considered ($P_0=900\,{\mathrm{mW}}$ and $P_0=1100\,{\mathrm{mW}}$), with an applied stretching time of two seconds and a monitoring of the relaxation behavior of two seconds. Because of the large biological variability, for each individual experiment the measurement has been taken with a minimum of about 100 cells.

In a first step, we identified some parameters and/or their ranges by comparison to published references.

An initial spherical deformable cell was created with a total of $N=642$ nodes (test runs indicated that further spatial refinement was not required). The cells were immersed in a medium with viscosity $\mu$ being close to that one of water. We approximate the cell-medium friction coefficients for each note needed to determine term (3) in Eq. (1) by $\gamma _{\mathrm{ns}} = \gamma _{\mathrm{liq}}/N= 6 \pi \mu R /N$. This approximation ensures that for a spherical cell of radius $R_{\mathrm{c}}$ modeled by *N* nodes at its surface, the friction coefficient is precisely as predicted by the Stokes equation.

Determination of the force terms (4) and (5) in Eq. 1 requires determination of the elastic modulus of the cell cortex. Because we did not have information on the elastic properties of the MDA-MB-231 cells, we adopted a nominal value for the elastic modulus of the cell cortex $E_{\mathrm{cor}} \sim 1\,{\mathrm{kPa}}$ based on different cell types (see Tinevez et al. 2009; Brugués et al. 2010). Applying Eqs. 7 and 10, which relate the elastic moduli to the parameters of the coarse-grained model of the CSK, the nominal cortical stiffness constants in the model are thus $k_{\mathrm{s}} \sim 4\times 10^{-4}\,{\mathrm{N}/\mathrm{m}}$, $k_{\mathrm{b}} \sim 1\times 10^{-17}\,{\mathrm{N/m}}$ (see Sect. 2.1, Eqs. 3 and 10). To determine the compression force of the cell represented by term (6) in Eq. (1), we need to know the cell compression modulus $K_{\mathrm{V}}$, which is still subject of debate. For instance, Delarue et al. (2014a, b) conclude from experiments with growing spheroids under pressure that cells are compressible with compression moduli of the order of 10 kPa. On the other hand, the Monnier et al. (2016) find individual cell compression moduli of several orders of magnitude higher (1 MPa) than the one reported above. Yet, Monnier et al. have measured this over short time period and accordingly they further state in their paper that on longer timescales, the compression modulus might differ from that value may due to adaptation of the cell. In the work of Tinevez et al. (2009), the cytoplasm compression modulus is estimated as $\pm ~2500$ Pa. Although this is not the compression modulus of the whole cell, it indicates that if cells are able to expel water through the aquaporins on longer timescales, this may be a good estimate of $K_{\mathrm{V}}$. We here adopted this value but we note that in the simulations for the optical stretcher, we did not find any significant influence on the results during the time course of the experiment when $K_{\mathrm{V}}$ was varied within $100\,{\mathrm{Pa}}$ to $10000\,{\mathrm{Pa}}$, see “Appendix 2”.

Term (7) of Eq. (1) represents the effect of bilayer compression modulus $K_{\mathrm{A}}$ (see Eq. 12). For pure lipid bilayers, about $0.2\,{\mathrm{N}/\mathrm{m}}$ has been reported, while in case of a plasma membrane of a cell, much lower values have been measured, likely due to invaginations and inclusions of other molecules in a regular cell membrane. In our model, we chose $K_{\mathrm{A}}=0.8\,{\mathrm{mN}/\mathrm{m}}$ (Brugués et al. 2010).

In the work of Ananthakrishnan et al. (2006), it was suggested that the cortical actin cytoskeleton is the main component determining the mechanical behavior of the cell. We follow this assumption, yet in order to simulate the experiment, we need to know the applied surface force vectors on each node generated by the deflecting laser beams (see Fig. 4c). The calculation of this force profile is described in “Appendix 3”.

In a second step, we identify the type of the viscoelastic elements connecting the surface nodes of the model cells (Fig. 5d) by comparing the results of the model simulations with those of the optical stretcher experiments with MDA-MB-231 cells. The characteristic relaxation behavior excludes purely elastic springs (Fig. 5). We first consider Kelvin–Voigt elements (KVE) between the surface nodes (Fig. 5d). Kießling et al. (2013) reported thermoelastic phenomena, whereby an increased laser power causes a transient temperature rise, which in turn modifies the viscoelastic properties of the cell. A thermal analysis of the used setup similar as in Kießling et al. (2013) indicated an ambient temperature increase of 20 to 25 K for a laser power from 900 to 1100 mW. The temperature rise and fall is very quick ($\sim {\hbox {ms}}$) in comparison with the duration of the experiment; hence, latency effects can be neglected. Because of the thermoelastic effects, we assumed in the model that the viscous properties (1) are changed by the laser power during the stretching stage to (i.e., $\gamma = \gamma (T)$), and (2) are all restored to their original value during release. A good fit for the $P=1100\,{\mathrm{mW}}$ was achieved by the values $k_{\mathrm{s}}=1.0 \times 10^{-4}\,{\mathrm{N/m}}$, $k_{\mathrm{b}}=2\times 10^{-17}\,{\mathrm{N/m}}$ as cortex elastic constants, $\gamma _{||} =\gamma _{\perp } = \gamma (T_{0})= 2.3\times 10^{-4}\,{\mathrm{Ns/m}}$ as nominal friction coefficient (see Fig. 5a) and by a temperature dependence of the friction coefficients of $\gamma (310K) / \gamma (T_{0}) = 0.29$ and $\gamma (315K) / \gamma (T_{0} )= 0.11$. The fitted elastic constants for the springs and bending thus correspond well to those obtained from the nominal material properties (see above). We then tested the assumption that the viscosity would scale with temperature as in the Arrhenius law (Kießling et al. 2013):

23 $$\begin{aligned} \frac{\gamma (T)}{\gamma (T_{0})} =\exp \left( \frac{E_{\mathrm{a}}}{R}\left( \frac{1}{T}-\frac{1}{T_{0}}\right) \right) , \end{aligned}$$

where $R =8.314 {\mathrm{J~mol}^{-1} \mathrm{K}^{-1}}$ and $T_{\mathrm{0}} = 290\,{\mathrm{K}}$. Using this equation, we obtained an optimal fit with the model calibrated friction coefficients (see Fig. 5c) using the value $E_{\mathrm{a}} =55\,{\mathrm{kJ~mol}^{-1}\,\mathrm{K}^{-1}}$, which is not too far to $E_{\mathrm{a}} =74\,{\mathrm{kJ~mol}^{-1}\,\mathrm{K}^{-1}}$ derived in Kießling et al. (2013). Neglecting thermoelastic effects, no simultaneous fit of the experimental results for both laser powers could be obtained: if the KVE model is calibrated such that an optimal fit is obtained for $P=1100\,{\mathrm{mW}}$; then, using the same constants we get a significant overestimation of the deformation for $P=900\,{\mathrm{mW}}$, and vice versa (result not shown). The influence of a change in cortex mechanical properties on the results is given in “Appendix 2”.

Although the stretching and the relaxation behavior are both qualitatively well captured if thermoelastic effects are accounted for, there is (1) a restoring effect of the cell shape in the simulations (solid like behavior) that is not observed in the time course of the experiment, and (2) the short axis deformation slightly exceeds the experimental one. This latter deviation [point (1)] might be a consequence of missing out an explicit representation of the internal cytoskeleton in the model (i.e., see Kubitschke et al. 2017), which might here result in a higher resistance to a movement of the cell surface perpendicular to the laser axis, but including an explicit representation of the cytoskeleton is not expected to remove the significant deviations of the model during the relaxation stage [point (1)]. For this reason, we capture a possible contribution from the fluid-like behavior of the cortical cytoskeleton (see, e.g., Brugués et al. (2010)), by adding an additional friction element in series with the spring in the Kelvin–Voigt element (with the choice of $\gamma (T_{0})= 10^{-5}\,{\mathrm{Ns/m}}$), resulting in a modified Maxwell model^8^ (MME) (see Fig. 5d). An optimal fit for the $P=1100\,{\mathrm{mW}}$ is now achieved using the elastic constants $k_{\mathrm{s}}=5 \times 10^{-4}\,{\mathrm{N/m}}$, $k_{\mathrm{b}}=2 \times 10^{-17}\,{\mathrm{N/m}}$, but with $\gamma (T_{0}) = 3\times 10^{-5}\,{\mathrm{Ns/m}}$ and $\gamma _{ME}(T_{0}) = 3\times 10^{-3}\,{\mathrm{Ns/m}}$ as overall damping parameter and for the Maxwell damping, respectively (Fig. 5b, d). The temperature dependence for the friction coefficients was chosen as obtained in the KVE model (Eq. 23). The two fits capture the relaxation behavior overall well and much better than the KVE model, with an apparent plastic deformation keeping the cell shape from its original value on the short time scale of the optical stretcher experiment.

In conclusion, in this section we have identified the basic biomechanical material properties of the DCM, namely the viscoelastic elements and its parameters, to quantitatively mimic optical stretcher experimental results. Our simulations indicate that the laser induced stretching and the subsequent fluid-like relaxation behavior after the stretching can be best mimicked assuming a modified Maxwell model for the elements (MMEs) representing the cortical cytoskeleton, with the additional assumption that the viscous properties decrease with increasing temperature as a result of the lasers. The KVE model overall results in less optimal fits but may still be useful in simulations, because the behavior of the cells, on longer timescales (minutes to hours) after the experiment is not a priori known and may exhibit active responses, likely leading to a restoring of the original cell shape (Gyger et al. 2014). Further refinements can be easily performed in the future to capture effects such as from the microtubule cytoskeleton (see Fig. 1).

#### Verification of cell adhesion forces in pull-off experiment

The DCM adhesion model used in this work has been able to predict the correct red blood cell spreading area on a surface given a certain adhesion strength. We have further validated this adhesion model on the level of the adhesive forces between two cells. In Chu et al. (2005), it was shown that JKR theory can be applied to living cells using micro pipette aspiration, providing a technique to determine the adhesion energy between cells experimentally. To verify that the resulting adhesive forces in our discretization are in the correct physical ranges, we have run several test simulations in which two adhered cells (with radius $R_1=R_2$) are mechanically separated by applying opposite forces to them. The separating force (also called pull-off force) is a well-known quantity in JKR theory for soft adhesive spheres and reads

24 $$\begin{aligned} F_{\mathrm{p}} = -\frac{3}{2}\pi W \hat{R}, \end{aligned}$$

where *W* is the adhesion energy per surface area (specific adhesion energy) and $\frac{1}{\hat{R}} =\frac{1}{R_1}+\frac{1}{R_2}$ (cf. Eq. (21)). On the other hand, adhesion between stiff spheres is best described by Derjaguin–Muller–Toporov (DMT) theory (Leckband and Israelachvili 2001), leading to a pull-off force of $F_{\mathrm{p}} = -2\pi W \hat{R}$. For vesicles, a theoretical analysis leads to $F_{\mathrm{p}} = -\pi W R$ (Leckband and Israelachvili 2001).

**Fig. 6:**
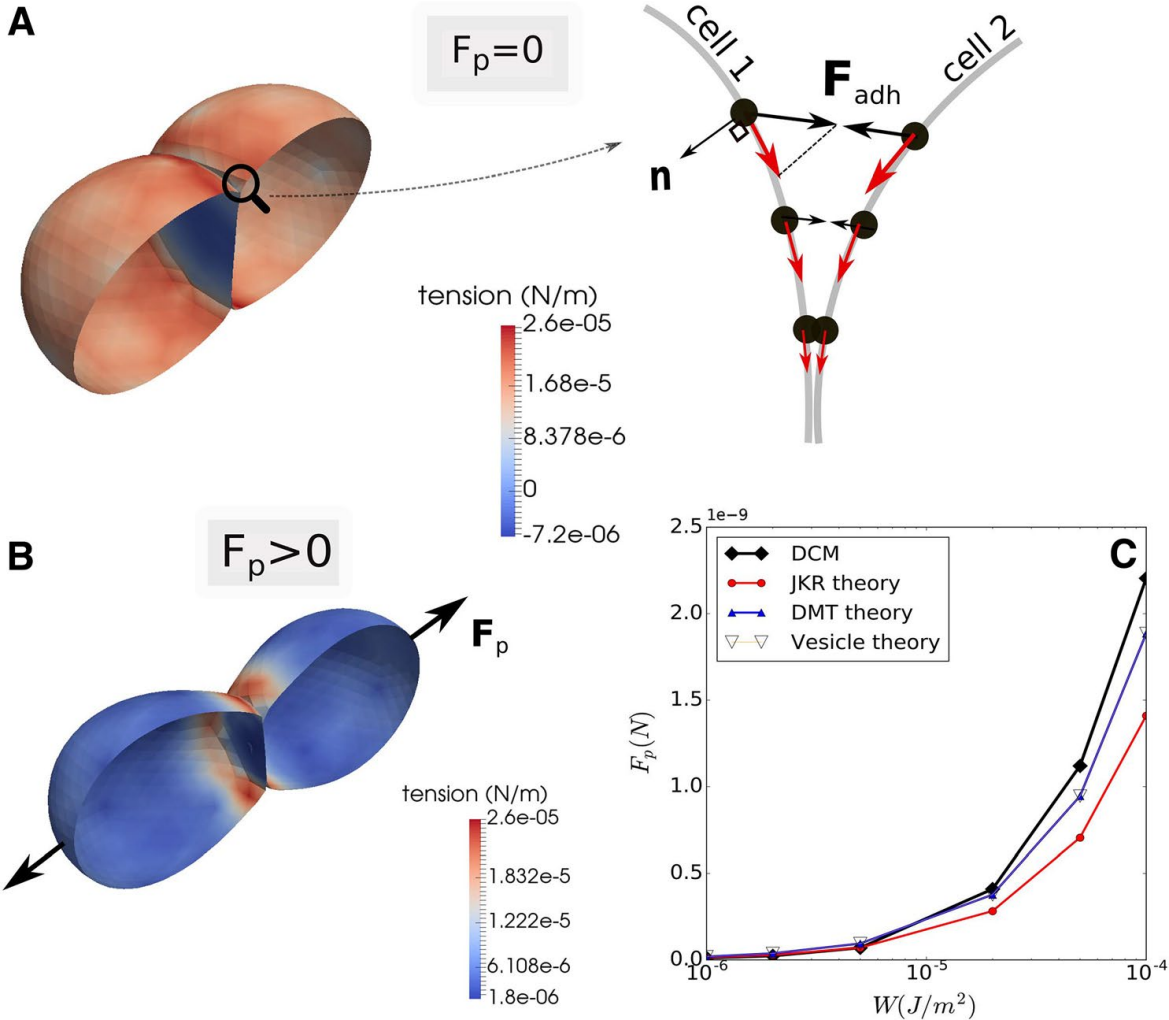
**a**, **b** Simulation snapshots of the experiment for determining pull-off force for adhering cells. The color coding is according to membrane stress. Stresses can be negative (compressive) in the common adhesive plane, indicated by the blue coloring (**a**, left). The 2D cartoon (**a**, right) shows the forces on the nodes ${\mathbf {F}}_{\mathrm{adh}}$. The resulting nodal force (red arrows) along the cell boundaries point toward the contact center. **b** Cell deformation during the pulling with net force $F_{\mathrm{p}}$. **c** Absolute values of pull-off forces obtained as function of the adhesion energy per unit surface area: DCM simulations compared to JKR theory, DMT theory, and vesicle theory. Video 1 of this in silico experiment is provided in the SI D

To measure the cell–cell adhesion force in our simulations, two cells in contact are first brought into contact and relaxed until they are in an equilibrium state. The adhesion energy is a genuine parameter in our model (see Sect. 2.1). Then, a force equal in magnitude is applied on each node of both cells into the direction perpendicular to the cell–cell contact, whereby the force on one cell is exerted in opposite direction than the force on the other cell. The orientation is chosen such that the contact is under tensile stress (negative load). The cells then move away from each other until a new equilibrium is reached (Fig. 6). The force at which the cells just separate defines the pull-off force. By performing several simulations following a binary search algorithm, we estimate the pull-off force. The operation is done for increasing adhesive forces and depicted in Fig. 6c. This shows that the simulated pull-off forces are slightly higher but close to the theoretical values as calculated from JKR theory, DMT theory, and vesicle theory. Overall, we can assume that the implementation of the adhesion energy and the choice of parameters in the model reproduce realistic magnitudes for the contact force.

A closer analysis of Fig. 6 (top) further reveals that tensions in the membrane (see Eq. 8) of the adhered cells in equilibrium are largely tensile except in the adhesive plane where they can be compressive. This is a direct consequence of the adhesive node–node forces at the boundary of a cell–cell contact, which generate forces (see red arrows in sketch, Fig. 6) on the nodes along the cell–cell interface toward the contact zone. This phenomenon is in agreement with experimental observations (Murrell et al. 2014). A very strong adhesion energy might even result in a buckling of the cell surfaces at the contact zone as we observed in test simulations, and as was suggested in Schwarz and Safran (2013).

It might be noticed that JKR and DMT theory locally average over the adhesion contacts, while in some applications one might like to keep the discrete form of the contacts. The latter is in principle possible by our model by associating different adhesion contacts with each node, and choosing the resolution of the cell surface (by the number of nodes per cell surface) high enough to capture the important aspects of the discreteness of focal contacts.

### Multicellular simulations: growth of small tumors and monolayers

In the past, multicellular spheroids (MCSs) and monolayers have often been used and tested as in vitro models for tissues (see, e.g., Sutherland 1988; Brú et al. 2003). Several authors have investigated the growth dynamics of these systems using agent-based models. In a number of communications, center-based models (CBM, see Sect. 2.3) have given basic insight how cell growth is affected by hypoxia and contact inhibition (Drasdo and Höhme 2005; Schaller and Meyer-Hermann 2005; Drasdo and Hoehme 2012). As mentioned before, CBMs generally suffer restrictions, such as poor shape representation.

Here, we employ our deformable cell model including cell division with regard to the aforementioned systems. In the growth dynamics of multicellular assemblies, important parameters are the strength of cell–cell adhesion responsible for the formation of multiple cell–cell contacts, and viscous cell–cell friction. To determine the cell–cell friction coefficients, we have simulated the relaxation behavior in an experiment whereby a spheroid is first compressed and subsequently the compression released (see “Appendix 1”). For long compression times, typical relaxation times of $\sim 5$ h have been reported for such experiments (Marmottant et al. 2009; Alessandri 2013). Hence, the spheroid relaxation times are much longer than the short relaxation timescales of the cells in the stretcher experiment, indicating that cellular re-organization processes not relevant in the optical stretcher experiments play a major role on the timescale of multicellular spheroid relaxation. For example, trypsinated cells in suspension round off, indicating that on timescales much longer than those probed in optical stretcher experiments, the cell relaxation behavior is governed by processes restoring a spherical cell shape, which is not correctly captured by the modified Maxwell element mimicking short term relaxation (Fig. 5d). Instead of further complexifying the viscoelastic elements to capture both the short and long-term relaxation behavior at the expense of elevating the computing time requirement,^9^ we here use the simple KV viscoelastic elements for the simulations at the timescale of cell growth and division. This allows to fit the internal friction coefficients and cell–cell friction coefficients such that the spheroid relaxation is approximately 5 h without prolonging the computing time (see Fig. 12b).

**Fig. 7:**
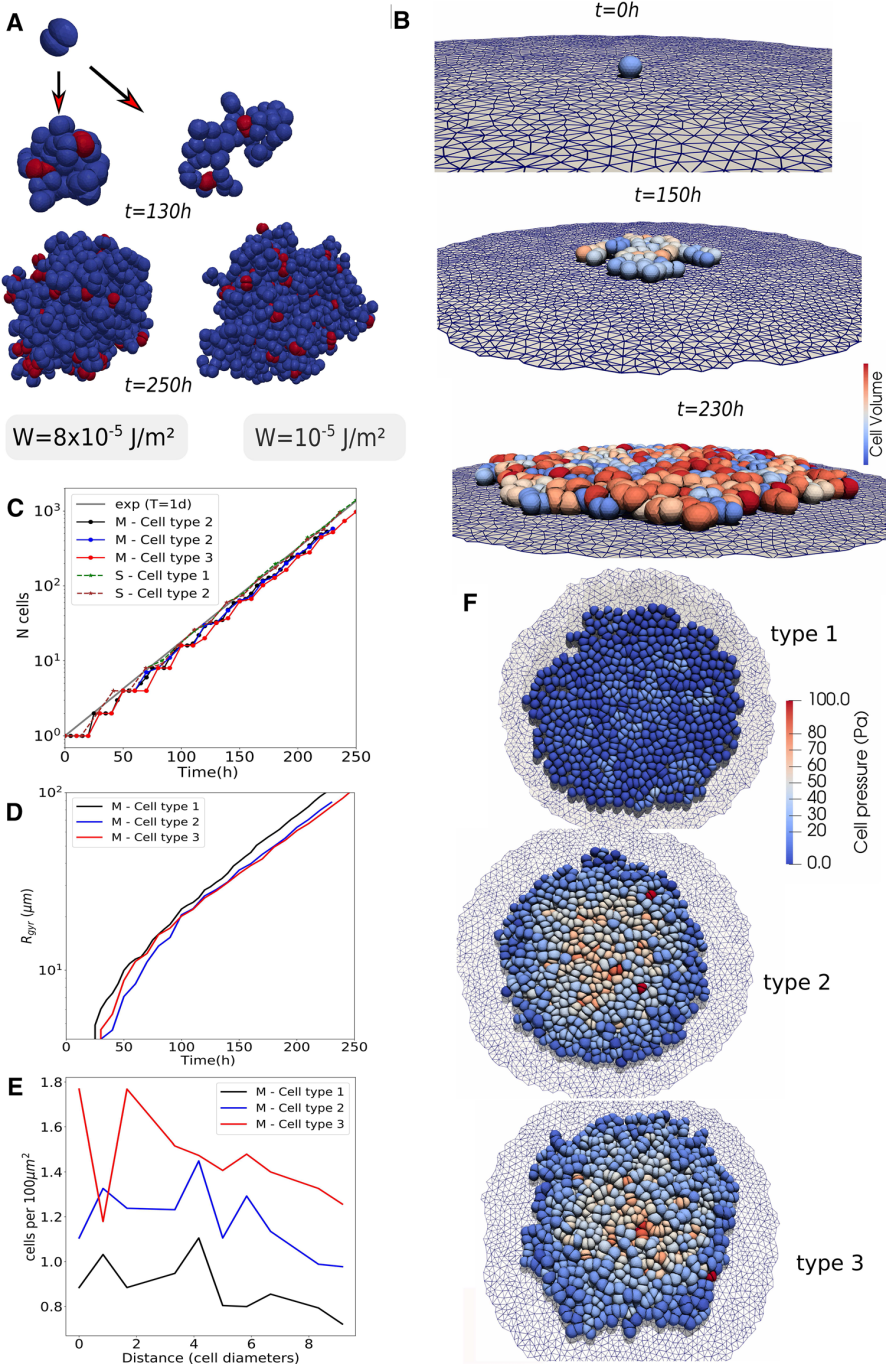
Simulation snapshots and time series for multicellular spheroid (MCS) and monolayer growth. **a** Spheroid growth with specific cell–cell adhesion energies $W = 1\times 10^{-5}\,{\mathrm{J/m}^2}$) and $W = 8\times 10^{-5}\,{\mathrm{J/m}^2}$). **b** Typical monolayer growth scenario (screenshots). **c**–**e** Kinetics of the cell population size *N*(*t*) (monolayers, MCS), radius of gyration $R_{\mathrm{gyr}}(t)$ (monolayers) and area density profile (number of cells per unit of substrate area) $\rho (r, N=1000)$ (monolayers). **f** Visualization of spatial pressure distribution in multicellular pattern for type 1–3 monolayers [the color encodes internal cell pressure (red: high, blue: low pressure)]. Parameters for monolayers: type 1: cell–cell specific adhesion energy $W \approx 0~\hbox {J/m}^2$, cell–substrate adhesion energy $W=10^{-5}J/m^2$, $D=10^{-15}~\hbox {m}^2/\hbox {s}$; type 2: cell–cell specific adhesion energy $W=10^{-5}~\hbox {J}/\hbox {m}^2$, cell–substrate adhesion energy $W=10^{-5}~\hbox {J/m}^2$, $D=10^{-16}~\hbox {m}^2/\hbox {s}$; type 3: cell–cell specific adhesion energy $W=5\times 10^{-5}~\hbox {J/m}^2$, cell–substrate adhesion energy $W=10^{-5}~\hbox {J/m}^2$, $D=10^{-16}~\hbox {m}^2/\hbox {s}$. Demonstrating video 2 and 3 of these in silico experiment are provided in the SI D

Both MCSs and monolayer simulations start from a single cell growing and dividing unlimited with a cell cycle time of 24 h. Each cell is represented by 162 nodes. For an overview of all the reference parameters, see Table 1. The simulations are run over several days, in which in total about 1000 cells are created (see time series of simulation snapshots in Fig. 7a, b for MCSs and monolayers, respectively). Two cases were simulated, one with the original specific adhesion energy, $W = 10^{-5}\,{\mathrm{J/m}^2}$, one with an increased specific adhesion energy, $W = 8\times 10^{-5}\,{\mathrm{J/m}^2}$. However, the cell population sizes were too small to objectify differences in the tumor spheroid shape despite the spheroid with $W = 8\times 10^{-5}\,{\mathrm{J/m}^2}$) looks slightly more compact compared to the other case, where intermittently, small “branches” form (Fig. 7a, right). At this cell population size, the number of cells grows exponential in time in both cases (Fig. 7c).

In a further step, we test our model for three different cases of monolayer growth. We first constructed a flat fixed adhesive surface, triangulated with a slightly larger mesh size than the one used for the cell surface triangulation. The simulation starts from one cell adhering to the surface (the reference adhesion energy per area is $W = 10^{-5}\,{\mathrm{J/m}^2}$). To study the effect of adhesive strength, we simulated monolayer growth scenarios for three different parameter combinations, denoted as type 1–3, with type 1: specific cell–cell adhesion energy $W=10^{-16}\,{\mathrm{J/m}}^2$, and relatively large diffusion constant $D=10^{-15}\,{\mathrm{m}}^2/{\mathrm{s}}$; type 2: specific cell–cell adhesion energy $W=10^{-5}\,{\mathrm{J/m}}^2$, and diffusion constant $D=10^{-16}\,{\hbox {m}}^2/{\hbox {s}}$; type 3: specific cell–cell adhesion energy $W=5\times 10^{-5}\,{\hbox {J/m}}^2$, and diffusion constant $D=10^{-16}\,{\hbox {m}}^2/{\hbox {s}}$ (Fig. 7c–f). Visually, no large difference between the spatial pattern for different parameter combinations can be seen. This is objectified by plotting the radius of gyration $R_{\mathrm{gyr}}(t)$ for the three different parameter combinations, which is a measure for the spatial cell spread. It is defined as $R_{\mathrm{gyr}} =\sqrt{(1/N)\sum _i{(\mathbf {x}_{i} -\mathbf {x}_{\mathrm{com}})^2}}$, where $\mathbf {x}_{i}$ and $\mathbf {x}_{\mathrm{com}}=(1/N)\sum _i\mathbf {x}_{i}$ are the center-of-mass positions of the individual cells and the whole multicellular cluster, respectively. There is no significant difference in $R_{\mathrm{gyr}}$ for the three different types. All three populations grow exponentially fast during the simulation time period. However, the pressure profile differs: At a population size of 1000 cells, the pressure in the center of the monolayer is largest for type 3, the smallest for type 1 and intermediate for type 2. For type 1, the cell–cell adhesion energy is almost zero while the micro-motility is the largest, which results in a maximum relaxation of compressive stress and lower cell density (Fig. 7e). The specific cell–cell adhesion energy for type 3 cells is higher than for type 2 cells while all other parameters are the same, resulting in a higher adhesion and hence a higher compression (highly jammed state) in the interior of type 3 cell populations compared to the interior of type 2 populations (Fig. 7e). The spatial profile shows a decrease from the center to the border, in qualitative agreement with earlier observations in center-based model simulations (e.g., Byrne and Drasdo 2009; Galle et al. 2005). Contrary to the former studies, the cell volume and pressure in DCM simulations can be calculated more precisely.

In summary, these simulations show the potential of the model to analyze in detail forces, shape and pattern formation in monolayers and spheroids. We emphasize that the aforementioned results are indicative and that much more and much longer simulations maybe required to come to strong conclusions. With the current implementation (single core, cell cycle time of 1 day), it takes roughly half an hour of CPU time to simulate a growing DCM spheroid from one cell to 10 cells; 12 h to reach 100 cells; and about a week to reach 1000 cells. In comparison, this task takes only 5 min for a CBM model. The cause for this discrepancy is the increased number of nodes, the increase data structure size per cell, and the lower time step needed to keep the DCM simulation stable. As a consequence, DCM simulations with larger cell numbers will need to rely on a parallelization of the code.

**Table 1:** Nominal physical parameter values for the model. An (*) denotes parameter variability meaning that the individual cell parameters are picked from a Gaussian distribution with $\pm 10 \%$ on their mean value. Note, that the contact friction parameters in the table denoted with a superscript "+" are to be multiplied by the respective contact area between the cell and the interacting structure in order to obtain the values inserted in the above equations i.e., γ_x_ = γ_x_^+^A, where γ_x_ denotes the friction parameters in the text for structure x in units of Ns/m and A the contact area. CR: Calibration Runs

Parameter	Symbol	Unit	Value	References
*Deformable cell model*				
Radius (undeformed)	$R_{\mathrm{c}}$	μm	8.8–12	Observation, Hoehme et al. (2010)
Cycle time*	$\tau$	h	24	Hoehme et al. (2010)
Cortex Young’s modulus	$E_{\mathrm{cor}}$	Pa	1000	Brugués et al. (2010)
Cortex thickness	$h_{\mathrm{cor}}$	nm	500	Observation
Cortex Poisson ratio	$\nu _{\mathrm{cor}}$	–	0.5	Tinevez et al. (2009)
Membrane area compression	$K_{\mathrm{A}}$	N/m	$0.8\times 10^{-3}$	Brugués et al. (2010)
Cell bulk modulus	$K_{\mathrm{V}}$	Pa	750–2500	Hoehme et al. (2010) and Tinevez et al. (2009)
Specific cell–cell adhesion energy	*W*	J/m^2^	$10^{-5} - 5\times 10^{-5}$	Hoehme et al. (2010), CR
Nodal friction	$\gamma _{\mathrm{int}}$	Ns/m	$1\times 10^{-4}$	CR
Cell–cell friction	$\gamma _{\mathrm{ext}}^{+}$	Ns/m^3^	$5\times 10^{10}$	Galle et al. (2005) and Buske et al. (2011), CR
Cell-ECM friction	$\gamma _{ECM}^{+}$	Ns/m^3^	$10^{8}$	Galle et al. (2005), CR
Cell-liquid friction	$\gamma _{\mathrm{liq}}^{+}$	Ns/m^3^	500	CR
Motility	*D*	m^2^/s	${10^{-16}}$	Drasdo and Hoehme (2012) and Hoehme et al. (2010)
*Center-based model*				
Radius (free suspension)	$R_{\mathrm{c}}$	μm	12	Hoehme et al. (2010)
Young’s modulus$^*$	*E*	Pa	450	Hoehme et al. (2010)
Poisson’s modulus	$\nu$	–	0.47	See $K_{\mathrm{V}}$, Hoehme et al. (2010)
Cell–cell friction	$\gamma _{\mathrm{ext}}^{+}$	Ns/m^3^	$10^{10}$	Galle et al. (2005) and Buske et al. (2011), CR
Cell-ECM friction	$\gamma _{ECM}^{+}$	Ns/m^3^	$5\times 10^{8}$	Galle et al. (2005), CR
Motility	*D*	m^2^/s	${10^{-16}}$	Drasdo and Hoehme (2012) and Hoehme et al. (2010)
Specific cell–cell adhesion energy	*W*	J/m^2^	$10^{-5} - 5\times 10^{-5}$	CR
*Lobule network*				
Radius lobule	$R_{\mathrm{lob}}$	μm	280	Hoehme et al. (2010)
Radius portal veins	$R_{\mathrm{pv}}$	μm	15	Hoehme et al. (2010)
Radius central veins	$R_{\mathrm{cv}}$	μm	10	Hoehme et al. (2010)
Radius sinusoids	$R_{\mathrm{sin}}$	μm	4.7	Hoehme et al. (2010)
Sinusoids Young’s modulus	$E_{\mathrm{sin}}$	Pa	600	Hoehme et al. (2010)
Sinusoids Poisson’s modulus	$\nu _{\mathrm{sin}}$	–	0.4	Hoehme et al. (2010)
Specific cell-sinusoid adhesion energy	*W*	J/m^2^	0	Hoehme et al. (2010)
Radius lesion	$R_{\mathrm{nec}}$	μm	150	Hoehme et al. (2010)

### Multicellular simulations: regeneration in a liver lobule

Finally, we come back to the introductory example of the regenerating liver lobule after CCl4-induced pericentral damage, and study whether the regeneration process with the DCM model would lead to different results than obtained with the CBM model (Hoehme et al. 2010). As the smallest repetitive physiological and anatomical unit is a liver lobule, our model simulations focus on this unit. A liver lobule has a central vein located approximately in its center that collects blood from the capillary (sinusoidal) network surrounding the central vein. The blood enters the liver lobule through 3–4 pairs of portal veins and hepatic arteries. Portal veins carry blood from the intestine to the liver contributing about 70% of the blood entering the liver lobule, and hepatic arteries carry oxygen-rich blood from the aorta to the liver. Hepatocytes are arranged around the capillaries. In the cut section perpendicular to the central vein, the lobular shape is approximately a hexagon. The distance from the central vein to the lobule border (radius of the lobule) is about nine cell diameters (Hammad et al. 2014). A typical 3D volume data sample obtained by processing of optical sections from confocal micrographs has a height of three cell layers. Between sinusoids and hepatocytes, there is an about 0.5 μm small space, named “Space of Disse” filled with ECM that mechanically stabilizes the sinusoidal network. The liver lobule micro-architecture ensures a maximum exchange area between hepatocytes and sinusoids for metabolites, thereby promoting metabolization in the hepatocytes. After overdose of the drugs CCl4 or acetaminophen (paracetamol, APAP) those cells expressing the Cytochrome P450 enzymes CYP2E1 and CYP1A2 metabolize these two drugs, which downstream can cause cell death. In healthy liver, CYP-expressing enzymes are localized in hepatocytes within an area fraction of about 40% around the central vein. At sufficiently high doses of CCl4 (or acetaminophen) the hepatocytes are killed leaving a central lesion with debris with maximal size at about 1–2 days after administration of the drug. About two days after the generation of the lesion, hepatocytes start to divide, reaching closure of the necrotic lesion and regeneration of the hepatocyte mass after about 6 days (Hoehme et al. 2010). The hepatocytes localized at and close to the border of the lesion proliferate by far the most, while proliferation is lower in the layers more distant to the lesion.

**Fig. 8:**
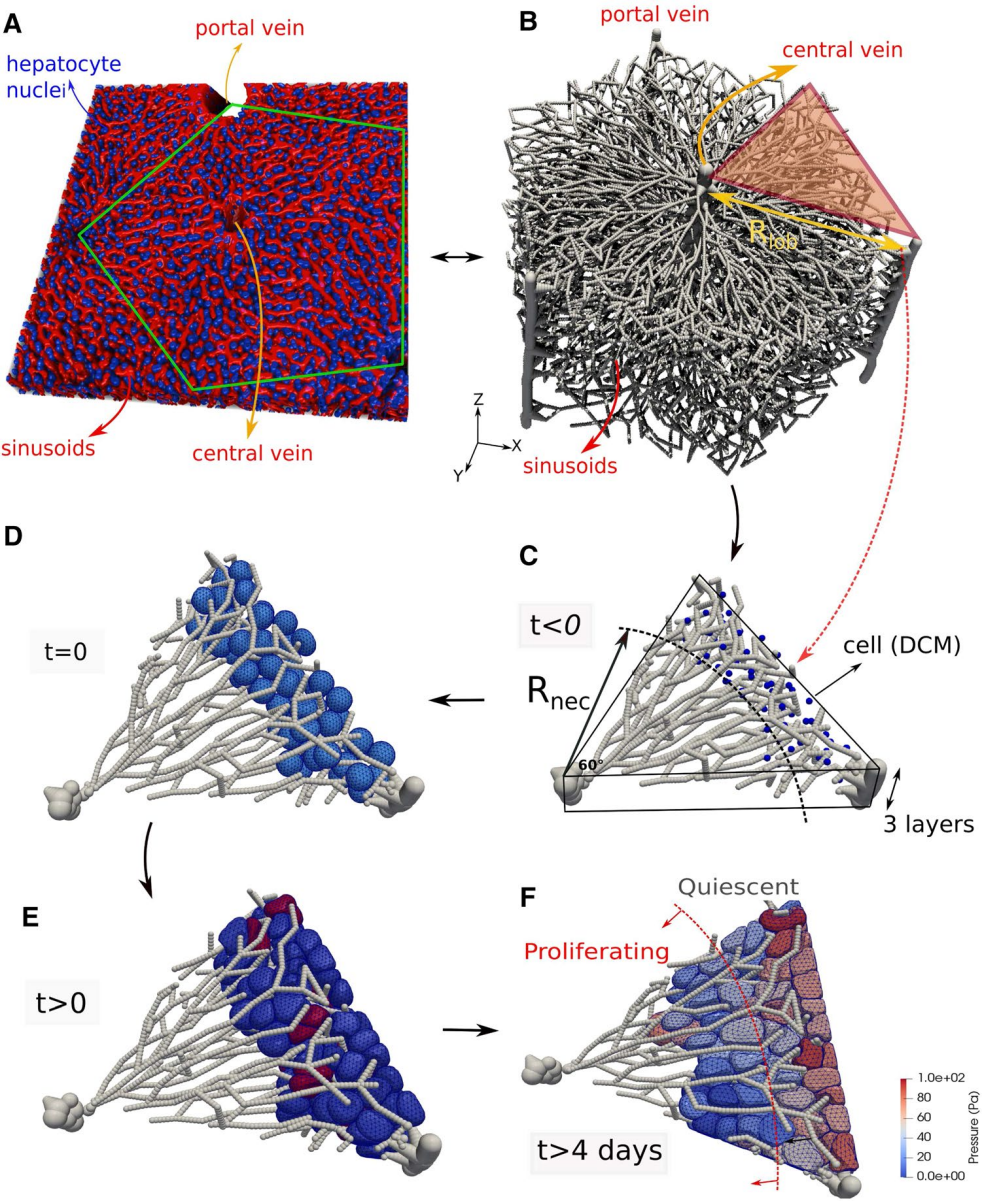
**a** Segmentation of hepatocyte nuclei (blue) and sinusoidal network (red) in liver tissue of mouse. Green lines outline a liver lobule substructure in the tissue. **b** Structure of the blood vessel network of a single lobule within the model. **c** Part of the network used for the simulations with the DCM. **c**, **d** Cells artificially growing up to realistic cell size (initialization). **e** The spatial-temporal proliferation pattern is imposed. The red cells are dividing. **f** Regeneration process the lobule, cells are colored according to internal pressure

The simulations are performed in a in statistically representative liver lobules, obtained by sampling of parameters quantifying the lobule architecture in confocal laser scanning micrographs (Hoehme et al. 2010). A statistically representative lobule has a hexagonal structure (Fig. 8a, b, *XY* plane) with a vertically (*Z*-axis) oriented central vein in its center and three portal veins and hepatic arteries in three of its six corners. Simulations with a center-based model revealed that cell proliferation alone, without directed migration of hepatocytes toward the central vein, is insufficient to explain the experimental observations (Hoehme et al. 2010). The mechanical pressure exerted by proliferating cells on their neighbors leading to pushing of cells and squeezing them into the spaces between the sinusoidal network was insufficient to close the lesion within the experimentally observed time period. In center-based models (CBM), cells are by construction usually relatively rigid as only moderate deviations of cell shape from their equilibrium shape in isolation can be captured by that model type. Furthermore, forces in standard center-based models are defined pairwise lumping compression and deformation forces usually together (see also Sect. 2.3.1). We here study if this rigidity might be responsible for the failure of regeneration in the experimentally observed time period.

As in Hoehme et al. (2010), we used a three-cell-thick lobule architecture representative of a mouse liver. The sinusoids and veins are approximated as a dense network of fixed overlapping spheres, each with radius equal to the vessel radii. The high density of overlapping spheres warrants a smooth interaction with the cells. The hexagonal shape allows for identification of 6 statistically identical “pie segments” together constituting a full lobule. As a full lobule is currently not amenable to simulations with the DCM because of the large computation times, we simulate regeneration in only one pie, representing 1/6 of a statistically representative liver lobule (Fig. 8c) At the outer boundaries of the lobule pie segment, the cells are pushed back by vertical planes that mimic the presence of the neighboring lobule parts (see Fig. 8b). In our model, we assume that vessels can interact with the cells, yet their positions remain static during the simulation (Hoehme et al. 2010). We perform both DCM and CBM simulations of the regeneration and quantitatively compare the differences. Both simulations start from identical initial conditions and configurations and have the same model parameters (see Table 1). A maximum equivalence of mechanical parameters for both model types is ensured (see Sect. 2.3).

We start from a multicellular configuration characteristic for the beginning of the regeneration process after CCl4 (or acetaminophen)-generated damage, where only hepatocytes localized at distance of at least $R_{\mathrm{nec}}$ from the central vein survive (see Fig. 8c, d, blue colored cells). Hepatocytes localized at distances smaller than $R_{\mathrm{nec}}$ from the central vein are assumed to be killed by the drug and removed from the system as those express the CYP-enzymes metabolizing CCl4, which downstream leads to cell death. At $t<0$, there are about 90 cells positioned in the free spaces between the sinusoids. An initial small radius is assigned to the hepatocytes, so that they initially do not touch the sinusoids. Next, the hepatocytes’ size is artificially increased, gradually generating contact with the sinusoids, until the hepatocytes reach a radius of $R_{\mathrm{c}} = 12\,{\upmu \mathrm{m}}$ (Hammad et al. 2014) and the system a mechanical equilibrium (Fig. 8d). The time at this starting configuration is set to $t=0$ as we here focus on the regeneration process (which is about 2–2.5 days after drug administration Hoehme et al. 2010).

During regeneration, those cells that have survived the intoxication enter the cell cycle to grow and divide with a rate depending on their distance from the necrotic lesion. During the regeneration process, they gradually move toward the central part, and eventually close the lesion and restore the lobule hepatocyte mass. Hepatocytes do not enter as individual, isolated cells (like mesenchymal cells) but in a collective movement as a sheet (with epithelial phenotype). In the simulations performed in Hoehme et al. (2010), the spatiotemporal proliferation pattern of the cells during regeneration from an experimental *BrdU* staining pattern was directly imposed to the cells. *BrdU* stains cells in S-phase. Here, we impose a similar but a slightly coarse-grained proliferation pattern (for simplicity and better comparability to CBM simulations), where cells are proliferating in a certain time window (Fig. 10a) and in a region smaller than 4–5 cell layers closest to the necrotic lesion. Outside this window, cells are assumed to be quiescent. Despite we do not simulate the entire lobule, the simulation results can readily be extrapolated to an entire lobule. By extrapolating the ratio of the cells numbers before intoxication and after regeneration with regard to the dimensions (Hoehme et al. 2010), the simulation results can be directly compared to that work.

**Fig. 9:**
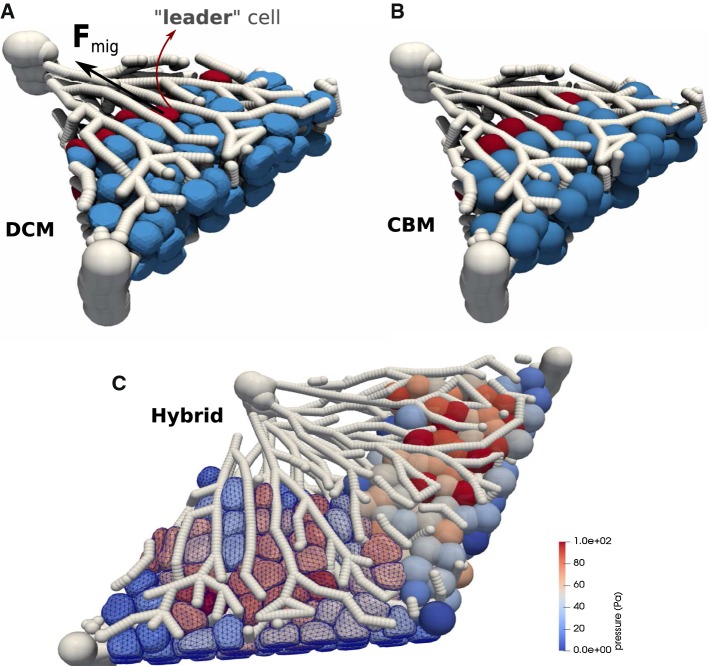
Simulation snapshots of the liver regeneration performed with the DCM (**a**) and CBM (**b**). **c** Simulation of CBM and DCM in hybrid mode. The color bar here is according to cell pressure. Videos 4 and 5 of the simulations are provided in the SI D

**Fig. 10:**
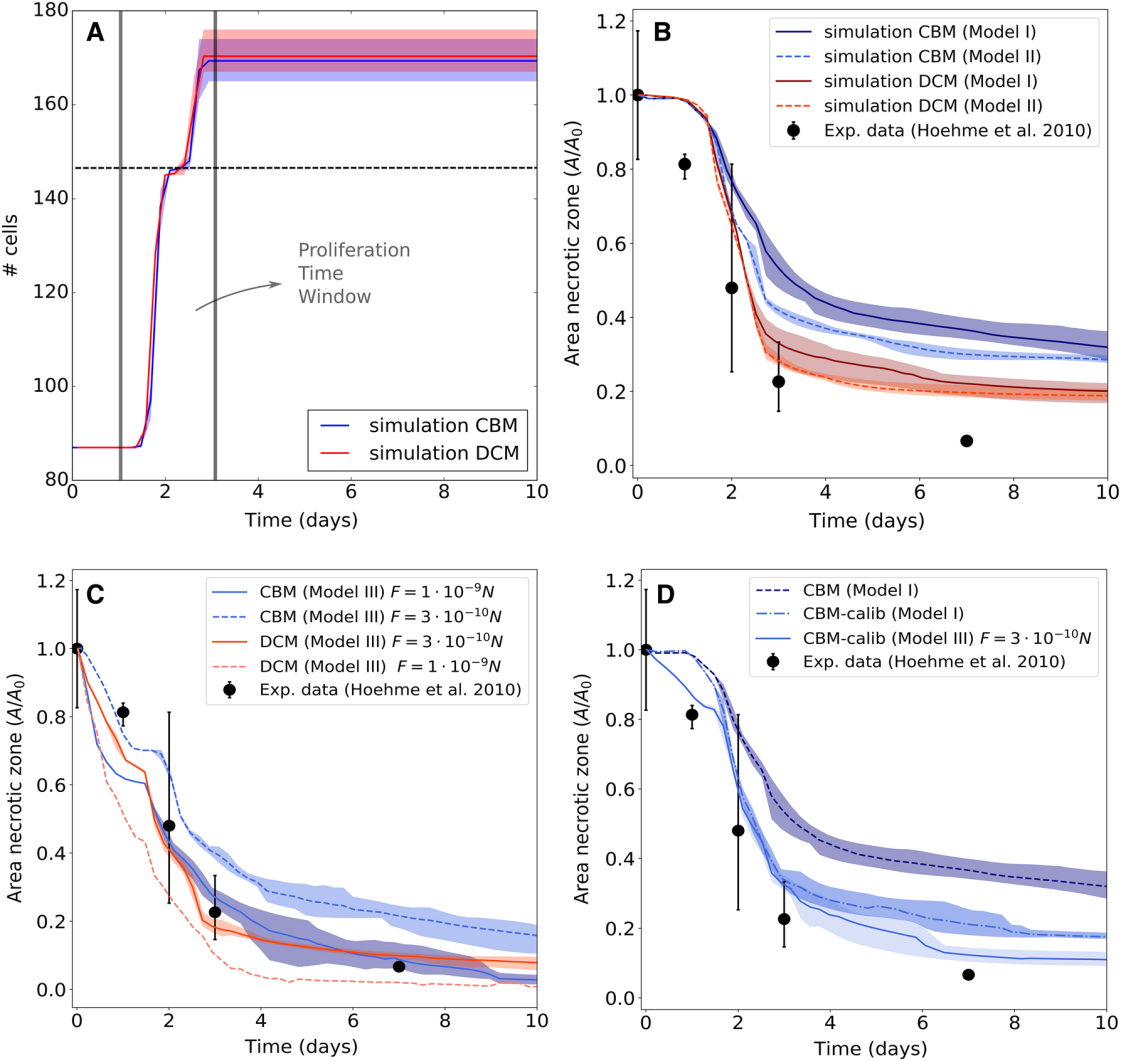
**a** Simulation (DCM and CBM) of the evolution of cell numbers during the time course of the simulation. **b** Simulated (DCM and CBM) relative area of the necrotic lesion during liver regeneration compared to data from Hoehme et al. (2010), assuming Model I or Model II. **c** Simulated (DCM and CBM) relative area of the necrotic lesion assuming Model III. **d** Simulated (CBM) relative area of the necrotic lesion using the calibration procedure for contact forces, assuming Model I or Model III. Each line is the average of 5 simulations with different random seeds, and the shadowed zone indicates maximum and minimum values

The cells gradually progress toward the central vein to close the lesion with the largest fraction of proliferating cells at the border to the lesion (the front of the expanding tissue) and smallest proliferation activity at the periportal field (Fig. 8f). During the simulations, various cell state variables such as pressure, volume, state and tissue properties (cell density, area of the necrotic zone) can be monitored (see Figs. 8, 9, 10)

In a next step, we study whether different hypotheses addressing the behavior of individual cells can explain the closure of the pericentral liver lobule lesion generated by toxic concentrations of CCl4 or acetaminophen. Guided by the choices in Hoehme et al. (2010), we distinguish between three model cases in which the cells differ by certain migratory or proliferation-related properties.

In the basic model (Model I), we assume the cells to be proliferating with daughter cells oriented in a random direction and active movement generated by an unbiased random force (controlled by the cell diffusion coefficient *D*).^10^ With this mechanism, the lesion closure speed^11^ is too small (see Fig. 10b), showing that this model is not in agreement with the experimental data for a time window of 10 days after intoxication, for both the DCM and the CBM. This is in line with the conclusion in Hoehme et al. (2010), stating that the pericentral lobule lesion does not completely close within the experimentally observed time period if the driving force for the closure is cell proliferation only. Nevertheless, the DCM simulations exhibit a closer agreement with the experimental data than the CBM. Potentially, this is due to the more realistic contact forces and cell shapes in DCM compared to the CBM, which permit deformable cells to adapt their shape by squeezing in between the sinusoids. The pairwise JKR-based contact force in CBM lumps force contributions originating from cell deformation and compression into one mathematical formula assuming the cell shape changes only slightly. As consequence, at large compression forces emerging as a result of massive cell proliferation as in liver regeneration, significant overlap among cells, and cells and blood vessels can occur. The large overlaps might lead to an underestimation of the total volume occupied by the cells and blood vessels and too small repulsion forces. This in turn critically slows down cell movement of cells that are pushed by proliferating cells toward the lesion, as the pushing forces also result from volume compression. In the DCM simulations, volume is explicitly tracked; hence, cells are pushed away stronger. At the same time, DCM cells can automatically adapt their shape and squeeze through the network. Both effects together speed up the cell displacement in DCM compared to CBM.

In the second model (Model II), we assumed that the during division the daughter cells align along the closest sinusoid, in Hoehme et al. (2010) named “hepatocyte-sinusoid alignment” (HSA) mechanism and identified as responsible order mechanism for the reconstitution of liver lobule micro-architecture. In Hoehme et al. (2010), healthy liver lobule micro-architecture was shown to be characterized by a maximal hepatocyte-sinusoid contact area, facilitating the molecular exchange area between hepatocytes and blood. However, contrary to the model of Hoehme et al., where cell polarity was introduced as anisotropic adhesive forces, we here do not impose cell polarity; HSA in combination with differential adhesion between hepatocytes and lack of adhesion between hepatocytes and sinusoids already result in a largely columnar order. Moreover, in Hoehme et al. (2010) HSA was combined with a directed migration of hepatocytes toward the central necrotic lesion, which we drop in Model II to test the effect of HSA only. Interestingly, with this “directed division” mechanism, we observe for both the CBM and the DCM a slight “speed-up” of the lesion closure, in line with the reasoning that cells get less obstructed by the sinusoids after division if aligned. Yet, an acceptable quantitative agreement is not obtained (Fig. 10b).

Finally, in the third model (Model III) we assume that cells migrate toward the central vein as a response to a morphogen gradient, whereby the morphogen source is the necrotic lesion. The directed migration manifests itself as an active force ${\mathbf {F}}^{\mathrm{mor}}$ in the equation of motion (see Sect. 2.1). Cell migration in liver is mediated by the extracellular matrix (ECM) scaffold, whereby the ECM is localized mainly in the Space of Disse (Mazza et al. 2015). In Hoehme et al. (2010), hepatocytes at the border of the necrotic lesion were observed to stretch filopodia into the necrotic lesion. Consequently, the migration force ${\mathbf {F}}^{\mathrm{mor}}$ is assumed to only act on the outer cells (here referred to as “leader” cells) and directed toward the central vein.

In Hoehme et al. (2010), adding directed migration forces toward the central vein to Model I was able to ensure complete lesion closure.

For the DCM simulations with Model III, we found an excellent agreement with the data if the migration force had a strength of $F^{\mathrm{mor}} \sim 0.3\,{\mathrm{nN}}$. Contrary, for the CBM simulations with the same model (Model III) a significantly larger force of $1\,{\mathrm{nN}}$ is needed to close the lesion, showing again the significant and important differences between the two model approaches at the quantitative level (Fig. 10c). In turn, simulations with DCM with the migration force chosen as the migration force needed in the CBM to close the lesion (i.e., $1\,{\mathrm{nN}}$) led to a too fast motion of the hepatocytes, hence a too fast closure of the lesion (Fig. 10c). This again confirms DCM cells adapt easier to the obstructions imposed by the environment. However, overall the magnitude of the migration force for both approaches is in agreement with experimental observations of migrating cells (Kim et al. 2015; Jacquemet et al. 2015). Specific measurements for regeneration after drug-induced damage have so far not been performed.

The simplified formalism in CBMs thus leads to unadapted cell shapes (Fig. 9a, b) and possibly quantitatively incorrect contact forces and cell density. In order to test the influence of the contact forces between the cells on the results, we applied our procedure to re-calibrate the CBM contact forces from the DCM (see Sect. 2.3.1 and “Appendix 1”). In the procedure we simulate a spheroid compression experiment. The contact stiffness of every CBM cell is adjusted depending on by how many neighbor cells it is surrounded and by the relative distances between the cell and its neighboring cells, to finally obtain the same contact forces as in the equivalent DCM simulation. As such, we obtain a calibrated CBM that takes the effect of multiple cell contacts into account. Using the calibrated CBM, we rerun the simulations for Model I and Model II. For Model I, the lesion is now closing faster and comparable to the DCM simulations. For Model III we now obtained a much better agreement if using the same migration force as in DCM (Fig. 10d). This overall shows the significant impact of quantitatively correct contact forces for quantitative simulations of the cell dynamics in the liver lobule. Simulations with center-based models may still be used to give valuable quantitative insight if the cell contact mechanics is corrected. The DCM thus permits to partially correct for inaccuracies of the CBM, and to serve as an instrument for verification simulations for selective parameter sets, whereby parameter sensitivity analysis simulations might now still be performed with the computationally much faster CBM. We conclude that with a more refined cell model as the DCM, the magnitude of the active migration force needed to close the central necrotic lesion after drug-induced pericentral damage is smaller than for the center-based model, but overall the same mechanisms, i.e., active hepatocyte migration toward the necrotic lesion and oriented cell division are necessary for closure of the lesion. This qualitatively confirms the conclusion made in Hoehme et al. (2010) based on simulations with a CBM. However, as we demonstrated, the differences between CBM and DCM simulations become smaller if the cell–cell interaction forces in the CBM are calibrated by simulations with the DCM.

Finally, and in line with this argument, we demonstrate that the two model approaches are capable of working in a hybrid mode. This is particularly useful if one can subdivide the total system in zones with a higher interest where more detail is required (high interest), and others where only approximated dynamics is needed (low interest) (see, e.g., Kim et al. (2007)). Here, a lower computational cost can be obtained if the zone of lower interest can be simulated using CBM. Thanks to the nature of the contact model of the DCM, we can run simulations where parts of the lobule are covered by a DCM while others by a CBM. In Fig. 9c, we considered two equal parts of the liver lobule where each part is covered by another model. At the interface, the model ensures that cells from DCM and CBM smoothly interact.

## Summary and conclusions

In this paper, our main goal was to study in how far model-based statements and interpretations depend on the level of detail in the representation of cell shape and mechanics. Our showcase was the regeneration of hepatocutes after drug-induced liver damage.

We have developed a computational model that permits to explicitly mimic the cell shape and subcellular details. The starting point was a previously introduced deformable cell model (DCM) that has been used to simulate realistic cell shapes in response to local internal and external forces. In this work, we have extended this model with cell proliferation and motility to perform multicellular simulations, with a model for cell growth and division. We demonstrated how the viscoelastic elements can be chosen to explain data at timescales differing by order of magnitudes, namely optical stretcher experiments to probe cell biomechanics on a timescale of seconds, as well as tissue regeneration occurring at timescales of several days.

An adequate choice of the rheological model for the individual subcellular elements (representing the outer cytoskeleton) and the calibration of parameters hereof is an essential aspect for acquiring realistic simulations. Starting from simulating deformation of MDA-MB-231 cells in an optical stretcher experiment, we have shown that commonly used linear spring damper elements like Kelvin–Voigt and Maxwell with proper adaptations may be sufficient to reproduce experimental data over a short time course. Overall, the Maxwell model approach seems to be more realistic in predicting cell relaxation behavior. In line with the analysis in Kießling et al. (2013), changes of friction coefficients in response to the ambient experiment temperature changes were necessary. Performing in silico pull-off experiments imposing increasing adhesion energies, we have further verified that the adopted cell contact model consistently reproduces realistic forces in cell–cell adhesive contacts. Here, the model shows a further potential for investigating local stress distributions in cell–cell adhesive bonds, which can be important to understand mechano-transduction processes.

**Fig. 11:**
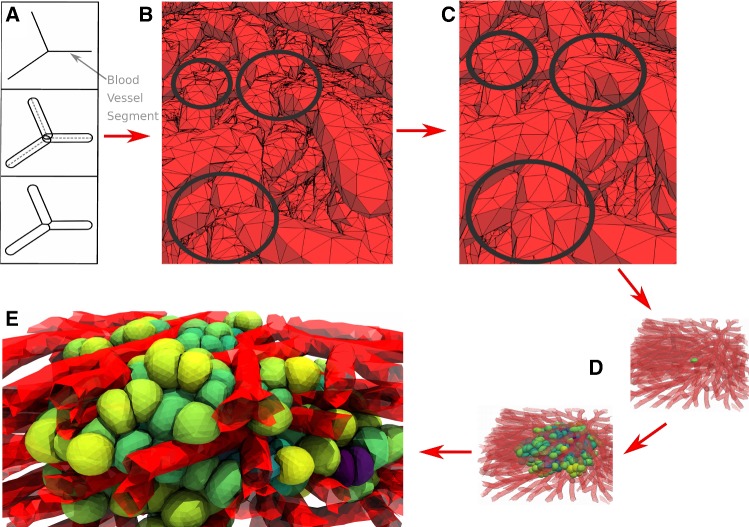
Simulation snapshots of a prototype model for liver tissue where the sinusoidal architecture and vessel shapes inferred from confocal micrographs. **a** From a graph structure of the blood vessel network, we can first approximate each blood vessel section as a cylinder with rounded ends. One can rotate and translate individual cylinders to match them with the position and direction of the original segment. **b** The cylinders are then meshed. We use the meshing algorithms available in the C++ CGAL library (The CGAL Project 2018). In a next step **c** we compute with CGAL the intersection of all vessel meshes and remove artifacts (indicated by circles) due to the intersection algorithm. Finally, we obtain a realistically and regularly meshed blood vessel network that allows interaction with, for example, growing DCM cells (**d**, **e**)

Cell growth and division occurs on much longer timescales than those probed by most experimental methods (optical stretcher or atomic force microscopy). Simulating cytokinesis explicitly by a contractile ring that splits the mother cell body into two approximately equal daughter cells, requires a re-meshing of the cell surface which turned out to be algorithmwise very complex. In our final algorithm, we let the cell in a first step grow to the twofold of its initial volume immediately after division. In the actual cell division step, two cell bodies are created which are inscribed in the original volume of the mother cell, increasing their volume until the volume of the mother envelop cell is precisely filled by its two daughter cells, after which the latter is removed.

The model allows to simulate in vitro experiments such as monolayers and spheroids growth from a single cell up to 1000 cells on a single processor of a standard PC in about one week time. Despite these relatively low cell numbers, we have here illustrated that model predictions with adhesion energy variations between cells and substrate may have different effects in monolayers as compared to spheroids.

Hoehme et al. (2010) demonstrated that agent-based models of the center-based type, mimicking cell–cell forces as forces between cell centers, are capable of giving valuable insights in liver regenerative processes. In particular, their simulations of a liver lobule regeneration showed that after intoxication, cell cycle re-entrance and cell division alone cannot explain the closure of lesions. By iterations between model simulations and experiments, they concluded that cells need active migrative forces in order to acquire a fully regenerated lobule in a realistic time course. However, the center-based model has no explicit notion of shape in case cells are densely packed as this is a case in a regenerating liver lobule. Deviations from its rest shape in isolation, typically a sphere, can only be approximately and statistically inferred from geometrical constructions as such as a partial Voronoi tessellation. Our simulations with the deformable cell model confirm this hypothesis, yet comparisons between our model and the center-based approach indicates some important quantitative differences, which we could use to identify model determinants that must be represented in sufficient detail.

The most critical differences between CBM and DCM simulations are:

1. In the DCM, cells adapt to their environment requiring lower migrative forces to close the gap as compared to their center-based counter parts. In the latter, large cell–cell overlaps exist and rigid cell shapes result in a lower closure speed or higher required migrative force to close the lesion.
2. Cell contact forces play an important role in the dynamics. Replacing the (standard) JKR contact force model in the CBM by new force relations obtained from simulated force probing experiments with the DCM largely removed the difference in the regeneration dynamics between CBM and DCM. We note that finding such a force might become almost impossible if, as in diseases such as fibrosis, the spaces between blood vessels and extracellular matrix structures are so narrow, that the cell body has to deform into a long thin object to squeeze through them. Another example where realistic contact forces for a CBM might not be found is in case of fluid-like movement of cells in cell sheets where cells move along each other despite high cell density. In such a type of movement, large deformation of cell shape from spheres might be necessary to permit cells moving, while at the same time, the volume of the cells is largely conserved. This combination—shape change at (almost) conserved volume—is an obstacle for center-based models.
3. DCM simulations result in more accurate cell shapes along the sinusoids as they adapt to the environment (cells do not overlap with sinusoids).

However, as demonstrated in this paper for a regenerating liver lobule, the DCM can be used to calibrate the CBM model and/or to simulate tissue organization processes in a hybrid mode. This may open possibilities to accomplish large scale tissue simulations where some parts are modeled at high spatial resolution models (DCM), while in other zones approximate models (CBM) are sufficient. In that case, the CBM, that performs well in case of moderate deformations and at moderate compressive stress becomes also more accurate under higher compressive stress. However, it is recommendable to verify in situations of high compression that the CBM simulation does indeed reproduce the results of DCM with sufficient accuracy at least for single parameter combinations.

In conclusion, we have shown that this highly detailed model approach may become mandatory to simulate tissue regeneration quantitatively, integrating subcellular information on cell shapes and biomechanics. For the future, we strive to run simulations directly starting from triangulated structures (representing cells, blood vessels, flexible or stiff structures, see prototype simulation explained in Fig. 11), which can be imported automatically from processed images (Friebel et al. 2015) without further approximations or assumptions about shape. For example, the deformable cell model could permit to run simulations directly from 3D image reconstructions of confocal micrographs, integrating the experimental staining information as, e.g., used in Morales-Navarrete et al. (2019) to stain hepatocyte apical and basal membranes as well as hepatocyte borders to compute a tensorial order parameter characterizing the order of hepatocytes in the tissue. Modifying the properties expressed by the markers as in a perturbation experiment will allow to mimic and predict realistic and detailed expected outcomes of real experiments, thus representing an in silico test model for experimental hypotheses.

The principle bottleneck is the high computational cost requiring distributed computing. Hence, large population DCM simulations will require code parallelization envisaged for the future. Prospectively, this and a prospective transfer onto quantum computers might open up enormous opportunities for the DCM toward an in silico parameter sensitivity testing to explain hypotheses, test hypotheses in the planning of new experiments and data acquisition to economize resources. Sensitivity analysis should then become automated (Jagiella et al. 2017).

### Electronic supplementary material

Below is the link to the electronic supplementary material.

Video 1. Simulation of a pull-off experiment with DCM to determine the relation between cell-cell adhesion energy and the forces needed to separate cells. The cells are first in adhesive equilibrium but then move in opposite directions due to opposing forces which are applied and evenly distributed on all the nodes. In this example, eventually these force break the adhesive bond between the cells.

Video 2. Simulation of spheroid growth with DCM starting from 1 cell up to 1000 cells.

Video 3. Simulation of monolayer growth with DCM starting from 1 cell. The substrate is assume to be adhesive and is modeled by a flat triangulated object.

Video 4. Simulation of the liver lobule regeneration process (hepatocytes moving towards the central vein) using DCM with a migrative force of 0.3 nN. The migration force is only applied to the {\ldquo}leader{\rdquo} cells (red colored). Only a part of the lobule is considered.

Video 5. Simulation of the liver lobule regeneration process using CBM with a migrative force of 1 nN. The migration force is only applied to the leader cells (red colored). Only a part of the lobule is considered.

## Figures and Tables

**Fig. 12:**
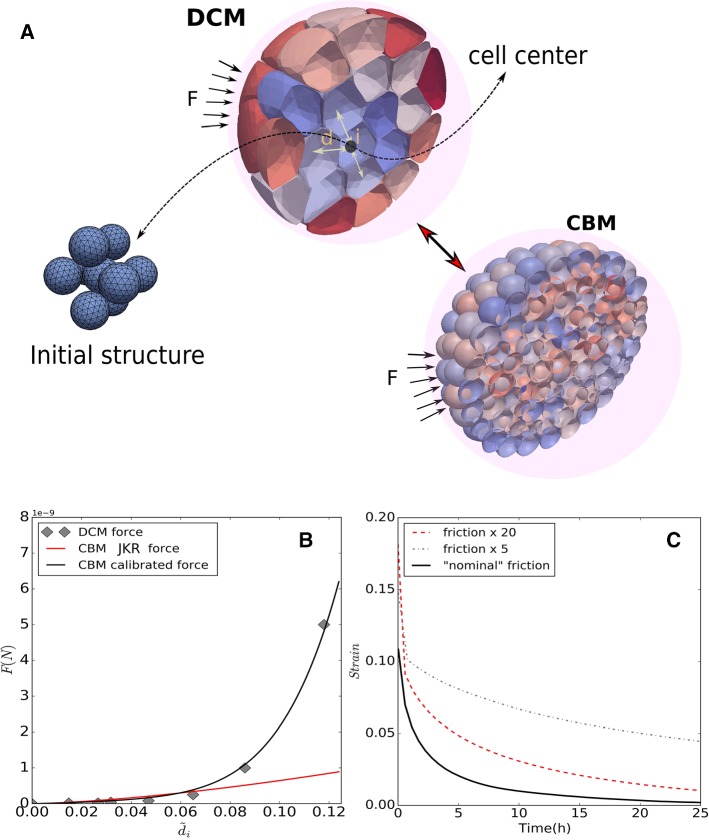
**a** Snapshot of a simulation of a small cell clump of DCM being compressed by isotropic forces. The distances *d* are computed between the cell centers and averaged. The equivalent CBM simulation is depicted below. **b** Force–distance data for a compression experiment simulated with DCMs, CBMs with using the JKR contact model, and CBMs using the calibrated contact model. **c** Simulated relaxation curves of a small spheroid performed with DCMs assuming different friction coefficients

**Fig. 13:**
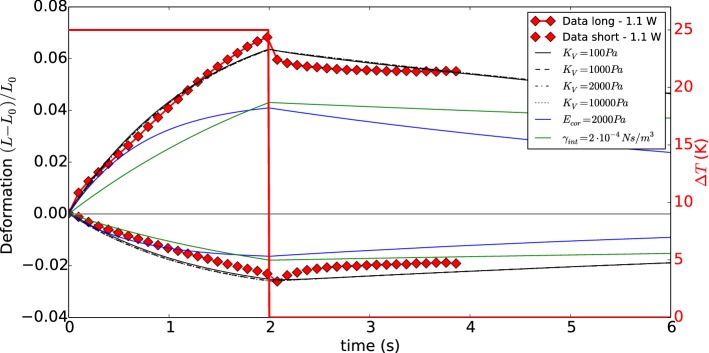
Comparison of the simulated MDA-MB-231 cell deformation with the experimental data in an optical stretcher, using a laser power $1100\,{\mathrm{mW}}$ (for clarity the laser power of $900\,{\mathrm{mW}}$ has been omitted). The model was solely fitted using a Kelvin–Voigt model (KVE) and assuming temperature dependent friction coefficients for the cytoskeleton. A parameter study was run for varying $K_{\mathrm{V}}$, showing very little effect of the cell compressibility for over 2 orders of magnitude. An increase in the cortex stiffness to $E=2000\,\mathrm{Pa}$ shows a significant decrease deformation of the cell, whereas a two-time increase in the cortex viscosity decreases the cell deformation and straightens out the curves

**Fig. 14:**
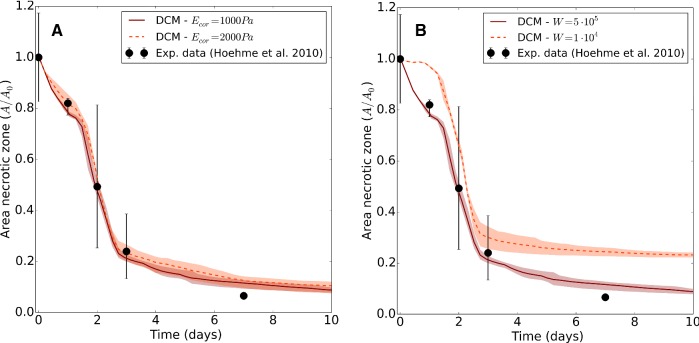
DCM parameter influence on the liver lobule lesion closing. **a** Simulation with two times high cell stiffness ($2000\,{\mathrm{Pa}}$) as compared to nominal value ($1000\,{\mathrm{Pa}}$). **b** Simulation with two times higher cell–cell adhesion ($10^{-4}\,{\mathrm{J/m}^2}$) as compared to nominal value ($5\times 10^{-5}\,{\mathrm{J/m}^2}$)

## Appendix 1: Determining DCM contact forces and friction coefficients

A small cell clump was used as in silico model to estimate the contact forces and relaxation in a cell aggregate. We considered a central cell neighbored by several others initially structured as a body centered cubic (Fig. 12a). The cell clump was then positioned in an imaginary rigid spherical capsule (see Van Liedekerke et al. 2019). The radius of the capsule was steadily and stepwise decreased, compressing the cells inside (Fig. 12a). The average distance function between the central cell and its contacting cells was computed as $\tilde{d}_{i} =\, <1 - d_{ij}/(R_i+R_j)>$ at every time step. This resulted in a forces-distance relation during the time course of compression that was used to calibrate the contact forces in the center-based model. In Fig. 12b, we have displayed the average force–distance relation during the experiment. The contact force in the center-based model shows a clear deviation as the distance gets smaller. This clearly shows that JKR or Hertz contact force models do not apply for high cell densities in CBM.

The full relaxation behavior of the spheroid is determined by the nodal friction coefficients, the viscous friction of the adherent cells, and the friction with the ECM. To measure relaxation, the cells are suddenly released from the encapsulation. This period for the cells to come back to their original state is a measure for the relaxation time of a spheroid, and is used to calibrate the friction coefficients. The relaxation state is quantified by a strain function measuring again the average distance function $\tilde{d}_i$ between the cells as a function of time. This is depicted in Fig. 12c. We choose the optimal friction coefficients so that the cell clump has a relaxation time of $\sim 5$ h as mentioned in Alessandri (2013); Marmottant et al. (2009). A model run with 5 times higher and 20 times friction is depicted to show a significant increase in relaxation times.

## Appendix 2: Sensitivity of the model to some mechanical parameters

In Sect. 3.1.1, we have estimated the mechanical parameters of the cell that gave a good agreement with the data for MDA-MB-231 cells. The model parameters that can directly influence the deformation of the cells upon external forces are the cell compression modulus ($K_{\mathrm{V}}$) the stiffness of the cortex ($E_{\mathrm{cor}}$), and the viscosity of the cortex ($\gamma _{\mathrm{int}}$). As it may be assumed that these parameters change along various cell types, we have performed a sensitivity study to the get an idea of their influence. In Fig. 13, we have depicted the results of simulations for a variation on their nominal values (see Table 1). A relatively small variation (factor of two) of cortex properties seem to greatly influence the deformability of the cell. Contrary, the bulk modulus of the cell does not seem to play any role herein.

Since we do not know all the mechanical parameters for hepatocytes, we partially re-used parameters of the MDA-MB-231 cells in the liver lobule simulations. As such, we may need to indicate how the mechanical cell properties influence the results on the lesion closing. Therefore as in the optical stretcher experiment, we run simulations with cell type with a variations on the parameters ($K_{\mathrm{V}}$, $E_{\mathrm{cor}}$, and $\gamma _{\mathrm{in}}$). In Fig. 14a, we have depicted the effect of a two times stiffer cortex, showing no large effect of this parameter, in contrast to the optical stretcher experiment. For a two times higher viscosity of the cortex, we found similar small effects (not shown). As in the optical stretcher experiment, a variation of the cell compressibility also did not show significant mutual differences (not shown).

However, as the lobule simulations include the cell–cell adhesion energy as additional parameter, we also run a set of simulations with a 2 times higher cell–cell adhesion. These indicate a significant effect of cell–cell adhesion on the lesion closing speed, as depicted in Fig. 14b.

## Appendix 3: Estimation of the surface forces on a cell during the optical stretcher experiment

Here, we give explain how the nodal forces on the cell are calculated from the optical laser beam. Therefore, we closely follow the approach detailed in Guck et al. (2001). The cells pass the optical stretcher experimental setup in suspension. The radius of the Gaussian laser beam is $R_{\mathrm{beam}} = 8.2\,{\mu \mathrm{m}}$ (Grosser et al. 2015). We can assume a ray optics approach, since the wavelength of the laser light, $\lambda = 1064\,{\mathrm{nm}}$ is much smaller than the diameter of our optical particles ($\approx 17 \mu m$).

Assume first a cube-like object which obstructs in the laser beam path. The laser beam enters the cube at the front side and leaves it at the back side. The incident momentum of the laser $p_i=nE/c$ (E is the energy of the incoming beam, *n* is the refractive index, and *c* is the speed of light), needs to be conserved at the interfaces of the medium with the cube. The surface picks up the difference of momentum $\Delta {p} = p_{\mathrm{incident}} + p_{\mathrm{reflected}} - p_{\mathrm{transmitted}}$ between the incident ray and the transmitted ray through the cube.

A resulting optical force acting due to momentum transfer for the frontal cube side is calculated using the Fresnel formulas and Newton’s second law (Guck et al. 2001):

25 $$\begin{aligned} F&= \frac{\Delta {p}}{\Delta {t}} = \frac{n_{\mathrm{m}} \Delta {E}}{c \Delta {t}} = \frac{n_{\mathrm{m}} Q P_0}{c}, \end{aligned}$$

where *E* is the energy of the photons, *c* is the speed of light, and $n_{\mathrm{m}}$ is the refractive index of the medium. The factor *Q* represents the transferred momentum ($Q=2$ for total reflection, $Q=1$ for total absorption) and total $P_0$ is the incident power. The same formula needs to be applied to the laser beam leaving the cube to the medium. The total force on one side of the cube at the laser axis due to the presence of two lasers in opposite direction can thus be calculated by summing up the transferred momenta at one side (Guck et al. 2000, 2001):

26 $$\begin{aligned} F_{\mathrm{total}}&= F_{\mathrm{front}} - F_{\mathrm{back}} \end{aligned}$$

27 $$\begin{aligned}&= \frac{n_{\mathrm{m}}Q_{\mathrm{front}}P_0}{c} -\frac{ n_{\mathrm{c}}Q_{\mathrm{back}}P_0}{c} \end{aligned}$$

28 $$\begin{aligned}&= [n_{\mathrm{m}}-(1-\mathcal {R})n_{\mathrm{c}}+\mathcal {R}n_{\mathrm{m}}]\frac{P_0}{c} - [n_{\mathrm{c}}-(1-\mathcal {R})n_{\mathrm{m}}+\mathcal {R}n_{\mathrm{c}}](1-\mathcal {R}) \frac{P_0}{c}. \end{aligned}$$

where $n_{\mathrm{c}}$ is the refractive index of the cube. The reflection $\mathcal {R}$ can be neglected for small incident angles, since according to the Fresnel equation:

29 $$\begin{aligned} \mathcal {R}&= \frac{\left( n_{\mathrm{c}} - n_{\mathrm{m}} \right) ^2}{\left( n_{\mathrm{c}} + n_{\mathrm{m}} \right) ^2} = 1.24 \times {}10^{-4} \approx 0. \end{aligned}$$

Hence, the total force on the cube interface simplifies to:

30 $$\begin{aligned} F_{\mathrm{total}}&= (n_{\mathrm{m}}-n_{\mathrm{c}})\frac{2P_0}{c}. \end{aligned}$$

This force acts always perpendicular to the object opposed to the incoming laser light. The applied stress in the direction of the laser beam on the cube is thus:

31 $$\begin{aligned} \sigma _{0}&= (n_{\mathrm{m}}-n_{\mathrm{c}})\frac{2P_0}{\pi R_{\mathrm{beam}}^2 c}. \end{aligned}$$

For $n_{\mathrm{m}} = 1.33$, $n_{\mathrm{c}}=1.36$, $P_0=1.1\text {W}$ and $c=3 \times {} 10^{8}\,{\mathrm{m/s}}$, we find $\sigma _0=1.05\,{\mathrm{Pa}}$.

Within our model, we need the applied stress for each triangular surface segment. However, the forces due to the laser beam are not equally distributed over the cell, as the cell is not a cube but has a spherical-like shape and the incident angle is not zero anymore but varies along the cell surface. A realistic overall stress profile can be assumed to be approximated by $\sigma (\alpha )=\sigma _0 \cos ^n \alpha$ with amplitude $\sigma _0$ and fitting parameter *n* (Ananthakrishnan et al. 2006). Here, $\alpha$ is the angle between the laser axes and the nodal position of the cell (see Fig. 4c). To determine the parameter *n*, we evaluate the ratio of the laser beam radius to cell radius:

32 $$\begin{aligned} \rho = \frac{R_{\mathrm{beam}}}{R_{\mathrm{cell}}} = \frac{8.2{\upmu \mathrm{m}}}{8.8{\upmu \mathrm{m}}} = 0.93 \end{aligned}$$

For a ratio of $\rho \approx 1$, *n* fits best when set to 2 (see, e.g., Guck et al. 2000, 2001; Ananthakrishnan et al. 2006). As we know how much surface area $A_{\mathrm{n}}$ each node on the cell represents, we can now calculate the forces on the DCM nodes by $F_{\mathrm{L}}(\alpha )=A_{\mathrm{n}}\sigma (\alpha )$, where $F_{\mathrm{L}}(\alpha )$ is oriented perpendicular to the cell surface at that node (Fig. 4c).

## Footnotes

1 From now on, we will use the term “DCM” uniquely for our triangulated deformable cell model.

2 In this paper, we assume that friction with ECM is isotropic and variations in cell shape do not alter the friction significantly.

3 Alternatively, the solution of Eq. (5) can be expressed analytically be means of an integrating factor leading to ${\text{F}}({\text{t}}) = {\text{e}}^{-{\text{k}}_{\text{s}} {\text{t}}/ \gamma_{\text{ME}}} \left[\int_{0}^{{\text{t}}} {\text{k}}_{{\text{s}}}{\text{v}}({\text{t}}^{\prime}){\text{e}}^{-{\text{k}}_{\text{s}}{\text{t}}^{\prime}/\gamma_{\text{ME}}}\, {\text{dt}}^{\prime} + {\text{C}} \right]$ where C is an integration constant. However, this equation would have to be equally discretized for integration.

4 We assume here a constant and homogeneous adhesion field. However experiments have pointed out that adhesion bounds are more point-like and can reinforce over time (Pawlizak et al. 2015). Although this consideration could be addressed with our model, it remains out of the scope of this paper.

5 $\hat{E}$ is given a large value compared to the cell Young modulus to prevent interpenetration of triangles.

6 A sub-simulation means that it runs in parallel and does not add up to the total simulation time (time is kept constant). The duration of a sub-simulation is very short, of the order of 10 s.

7 Note that $\tilde{d}_{ij}$ would correspond to the sphere-sphere overlap in the standard CBM.

8 Because of the presence of bending elasticity and resistance against area expansion of the cell plasma membrane, the overall behavior is not purely fluid-like but may rather be regarded as a Standard Linear Solid (SLS). However, these effects only appear on much longer timescales in the simulations.

9 The long-term relaxation into a spherical shape might be mimicked by implementing an additional spring with small spring constant in the MME element.

10 The assumptions in Model I are equivalent to those in Model 1 in Hoehme et al. (2010)

11 We define here the lesion radius as the average distance from the central vein to the closest cell in every layer whereby we consider the 3 closest layers to the lesion.
